# Supplementary material for: Carborane-Containing Hydroxamate MMP Ligands for the Treatment of Tumors Using Boron Neutron Capture Therapy (BNCT): Efficacy without Tumor Cell Entry
Source: Int J Mol Sci. 2023 Apr 9;24(8):6973. doi: 10.3390/ijms24086973 (PMC10139035; doi:10.3390/ijms24086973)
Supplement: Supplementary file 1 [file ijms-24-06973-s001.zip › ijms-2138099-supplementary.pdf]

SUPPORTING INFORMATION

Carborane-Containing Hydroxamate MMP Ligands for the Treatment of Tumors using Boron Neutron Capture Therapy (BNCT): Efficacy Without Tumor Cell Entry

Sebastian Flieger[1], Mao Takagaki[2], Natsuko Kondo[3], Marlon R. Lutz, Jr.[1], Yash Gupta[4], Hiroki Ueda[3], Yoshinori Sakurai[3], Graham Moran[1], Prakasha Kempaiah[4], Narayan Hosmane[5], Minoru Suzuki[3], and Daniel P. Becker[1*]

[1]Department of Chemistry and Biochemistry, Loyola University Chicago, Chicago, IL, 60660, USA

[2]Research Center for Nuclear Physics, Osaka University, 10-1 Mihoga-oka, Ibaraki-city, Osaka 567-0047, JAPAN

[3]Particle Radiation Oncology Research Center, Institute for Integrated Radiation and Nuclear Science, Kyoto University, 2-1010 Asashiro-Nishi, Kumatori, Sennan-gun, Osaka 590-0494 JAPAN

[4]Department of Medicine, Infectious Diseases, Mayo Clinic, Jacksonville, Florida, USA

[5]Department of Chemistry and Biochemistry, Northern Illinois University, DeKalb IL 60115, USA

*Correspondence: dbecke3@luc.edu

Table of Contents

1. HPLC of Closo-carborane complex from (R)-tert-Butyl 2-(4-Methoxyphenylsulfonamido-N-(prop-2-yn-1-yl))-3-methylbutanoate **8** S4
2. ^{1}H NMR of Closo-carborane complex from (R)-tert-Butyl 2-(4-Methoxyphenylsulfonamido-N-(prop-2-yn-1-yl))-3-methylbutanoate **8** S5
3. ^{13}C NMR of Closo-carborane complex from (R)-tert-Butyl 2-(4-Methoxyphenylsulfonamido-N-(prop-2-yn-1-yl))-3-methylbutanoate **8** S6
4. HRMS of Closo-carborane complex from (R)-tert-Butyl 2-(4-Methoxyphenylsulfonamido-N-(prop-2-yn-1-yl))-3-methylbutanoate **8** S7
5. HPLC of Carboxylic Acid Closo-Carborane **9** S8
6. ^{1}H NMR of Carboxylic Acid Closo-Carborane **9** S9
7. ^{13}C NMR of Carboxylic Acid Closo-Carborane **9** S10
8. HRMS of Carboxylic Acid Closo-Carborane **9** S11
9. HPLC of THP-Protected Hydroxamate Closo-Carborane **10** S12
10. ^{1}H NMR of THP-Protected Hydroxamate Closo-Carborane **10** S13
11. ^{13}C NMR of THP-Protected Hydroxamate Closo-Carborane **10** S14

12. HRMS of THP-Protected Hydroxamate Closo-Carborane **10** S15-16

13. HPLC of Hydroxamic Acid Closo-Carborane **3** S17

14. ^{1}H NMR of Hydroxamic Acid Closo-Carborane **3** S18

15. ^{13}C NMR of Hydroxamic Acid Closo-Carborane **3** S19

16. HRMS of Hydroxamic Acid Closo-Carborane **3** S20-21

17. HPLC of Methyl (*R*)-2-((4-methoxy-*N*-(pyridine-3-ylmethyl) phenyl) sulfonamido) pent-4-ynoate **(14)** S22

18. ^{1}H NMR of Methyl (*R*)-2-((4-methoxy-*N*-(pyridine-3-ylmethyl) phenyl) sulfonamido) pent-4-ynoate **(14)** S23

19. ^{13}C NMR of Methyl (*R*)-2-((4-methoxy-*N*-(pyridine-3-ylmethyl) phenyl) sulfonamido) pent-4-ynoate **(14)** S24

20. HRMS of Methyl (*R*)-2-((4-methoxy-*N*-(pyridine-3-ylmethyl) phenyl) sulfonamido) pent-4-ynoate **(14)** S25-26

21. HPLC of (R)-2-((4-methoxy-N-(pyridin-3-ylmethyl)phenyl)sulfonamido)pent-4-ynoic acid **(15)** S27

22. ^{1}H NMR of (R)-2-((4-methoxy-N-(pyridin-3-ylmethyl)phenyl)sulfonamido)pent-4-ynoic acid **(15)** S28

23. ^{13}C NMR of (R)-2-((4-methoxy-N-(pyridin-3-ylmethyl)phenyl)sulfonamido)pent-4-ynoic acid **(15)** S29

24. HRMS of (R)-2-((4-methoxy-N-(pyridin-3-ylmethyl)phenyl)sulfonamido)pent-4-ynoic acid **(15)** S30-31

25. HPLC of 4-methoxy-N-((2R)-1-oxo-1-(((tetrahydro-2H-pyran-2-yl)oxy)-l2-azaneyl)pent-4-yn-2-yl)-N-(pyridin-3-ylmethyl)benzenesulfonamide **(16)** S32

26. ^{1}H NMR of 4-methoxy-N-((2R)-1-oxo-1-(((tetrahydro-2H-pyran-2-yl)oxy)-l2-azaneyl)pent-4-yn-2-yl)-N-(pyridin-3-ylmethyl)benzenesulfonamide **(16)** S33

27. ^{13}C NMR of 4-methoxy-N-((2R)-1-oxo-1-(((tetrahydro-2H-pyran-2-yl)oxy)-l2-azaneyl)pent-4-yn-2-yl)-N-(pyridin-3-ylmethyl)benzenesulfonamide **(16)** S34

28. HRMS of 4-methoxy-N-((2R)-1-oxo-1-(((tetrahydro-2H-pyran-2-yl)oxy)-l2-azaneyl)pent-4-yn-2-yl)-N-(pyridin-3-ylmethyl)benzenesulfonamide **(16)** S35-36

29. HPLC of THP-protected 1,4-Click Isomer **18** S37

30. ^{1}H NMR of THP-protected 1,4-Click Isomer **18** S38

31. ^{13}C NMR of THP-protected 1,4-Click Isomer **18** S39

32. HRMS of THP-protected 1,4-Click Isomer **18** S40-41

33. HPLC of Hydroxamic Acid 1,4-triazole **4** S42

34. ^{1}H NMR of Hydroxamic Acid 1,4-triazole **4** S43

35. ^{13}C NMR of Hydroxamic Acid 1,4-triazole **4** S44

36. HRMS of Hydroxamic Acid 1,4-triazole **4** S45-46

37. Docking Figure **S1a** S47

38. Docking Figure **S1b** S48

39. Docking Figure **S2a** S49

40. Docking Figure **S2b** S50

41. Docking PDB Files S51

42. Cytotoxicity of Compound **3** (SF-4-6A) S52-53

43. Drug Water Solubility Raw Data (Figures S3-S6) S54-61

44. Figure S7a/b. Survival fraction in U87 MG cells and in SCC7 cells S62

45. Figure S8. Boron concentrations in SCC7 cells and U87 cells by ICP-OES S63

HPLC of Closo-carborane complex from (R)-tert-Butyl 2-(4-Methoxyphenylsulfonamido-N-(prop-2-yn-1-yl))-3-methylbutanoate **8.**

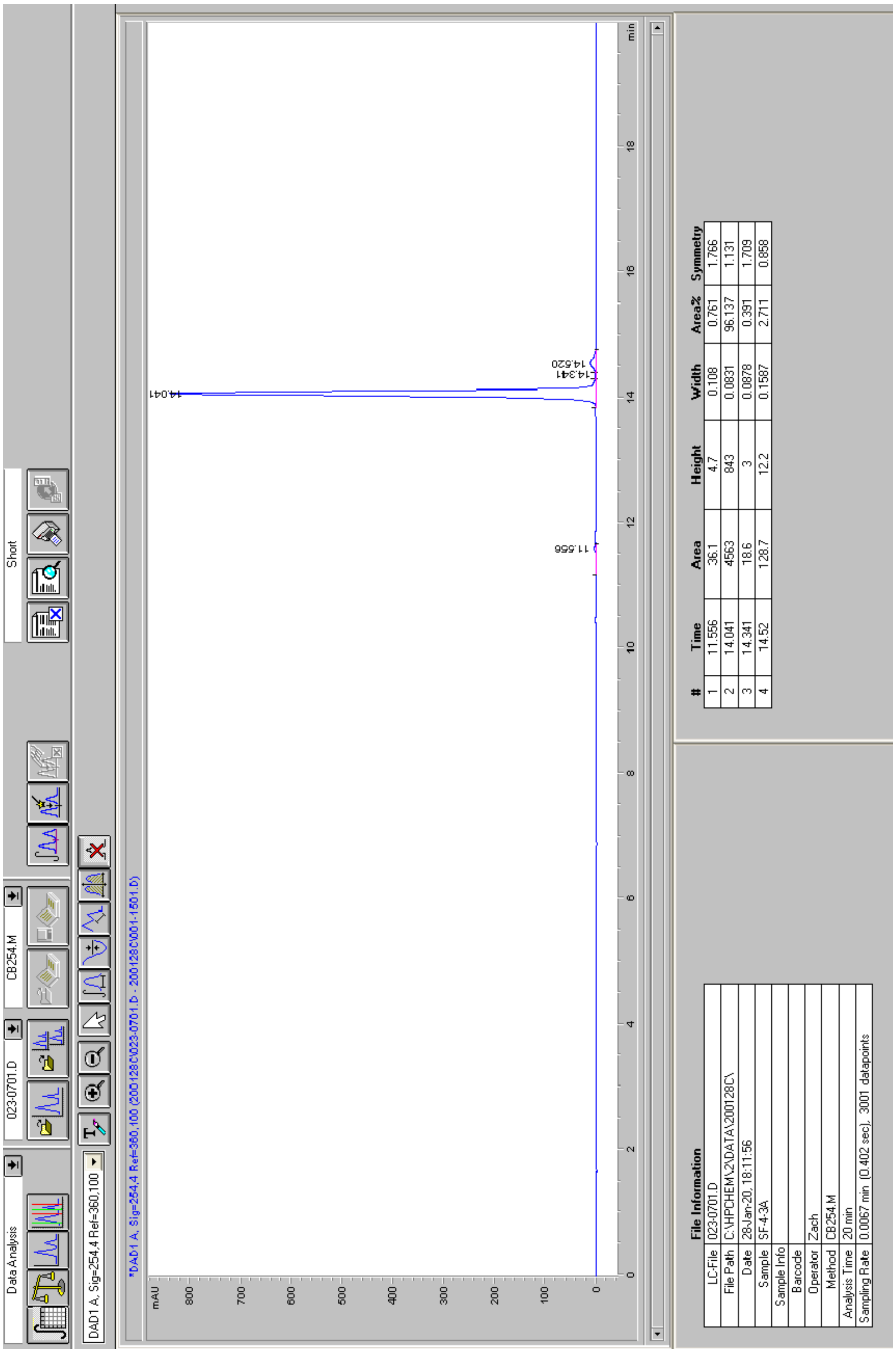

^{1}H NMR of Closo-carborane complex from (R)-tert-Butyl 2-(4-Methoxyphenylsulfonamido-N-(prop-2-yn-1-yl))-3-methylbutanoate **8.**

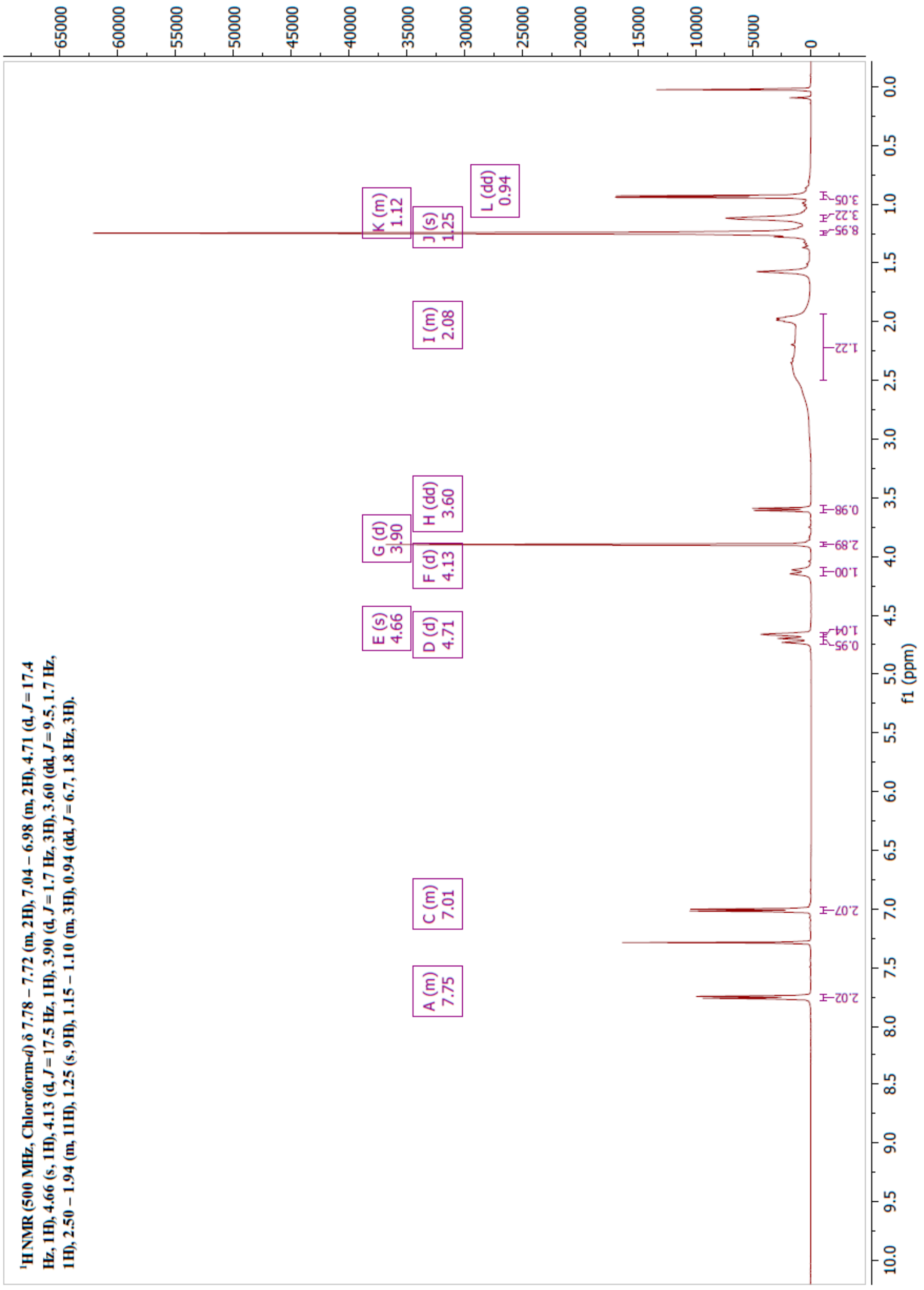

^{13}C NMR of Closo-carborane complex from (R)-tert-Butyl 2-(4-Methoxyphenylsulfonamido-N-(prop-2-yn-1-yl))-3-methylbutanoate **8.**

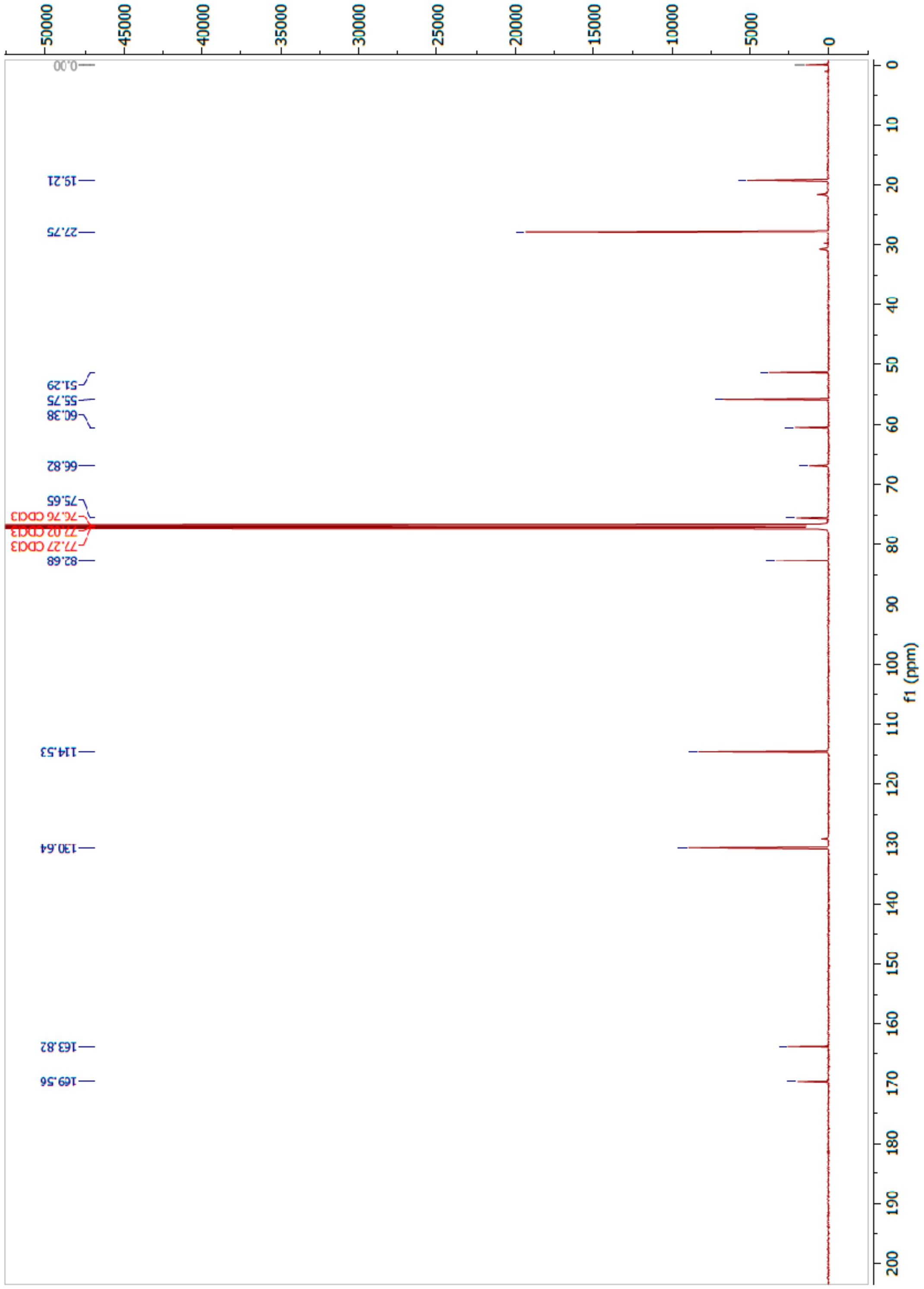

HRMS of Closo-carborane complex from (R)-tert-Butyl 2-(4-Methoxyphenylsulfonamido-N-(prop-2-yn-1-yl))-3-methylbutanoate **8.**

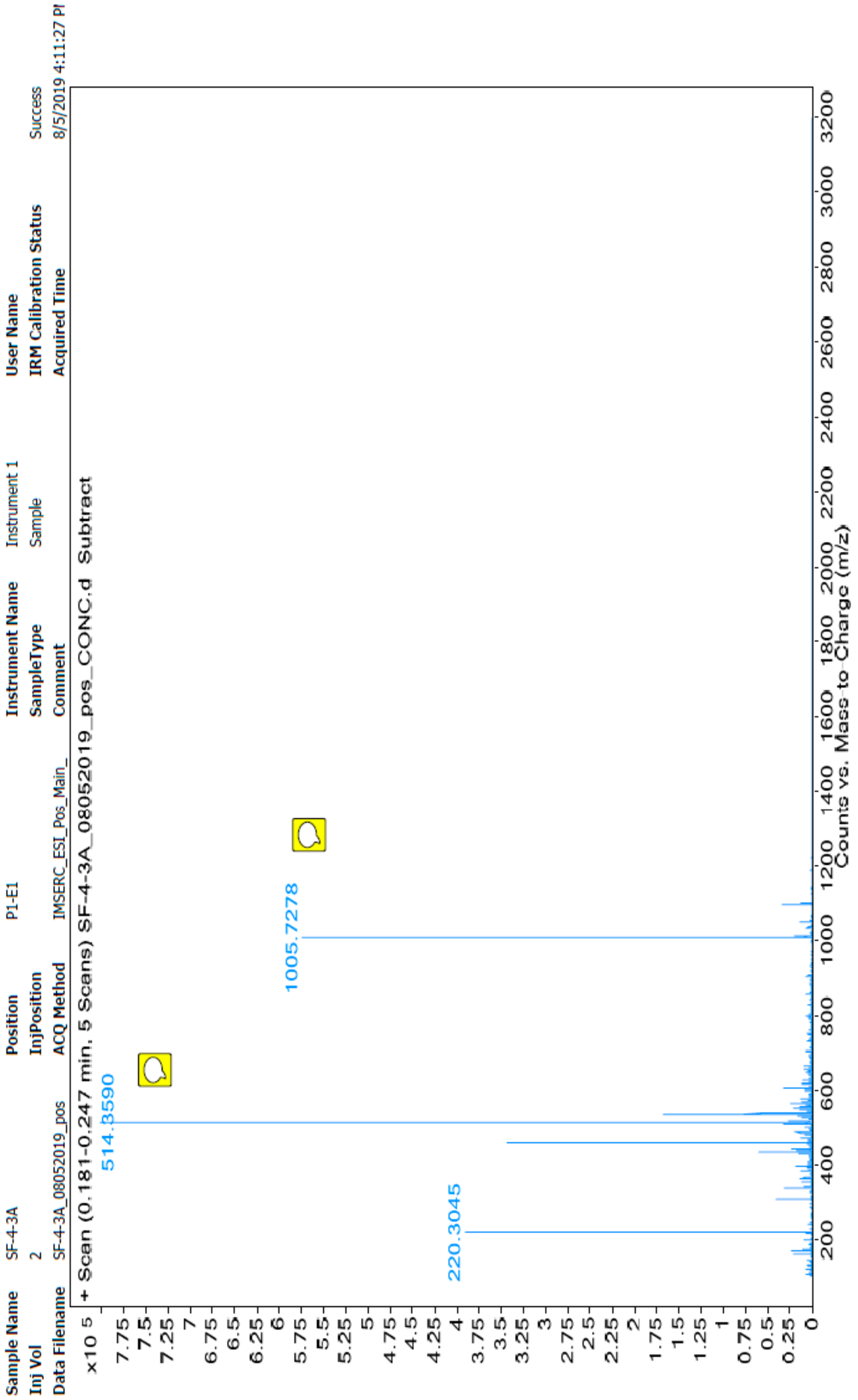

HPLC of Carboxylic Acid Closo-Carborane **9.**

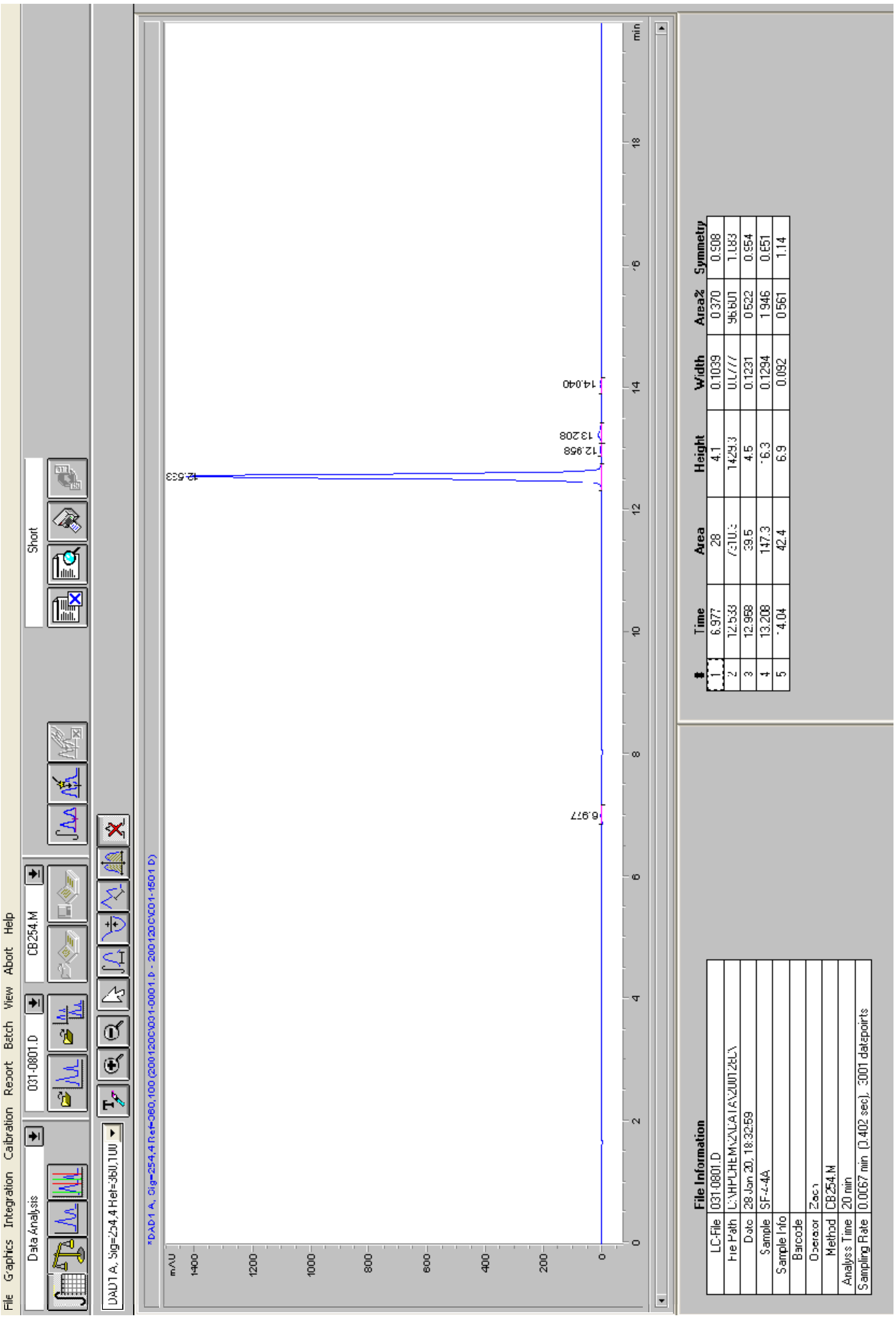

^{1}H NMR of Carboxylic Acid Closo-Carborane **9.**

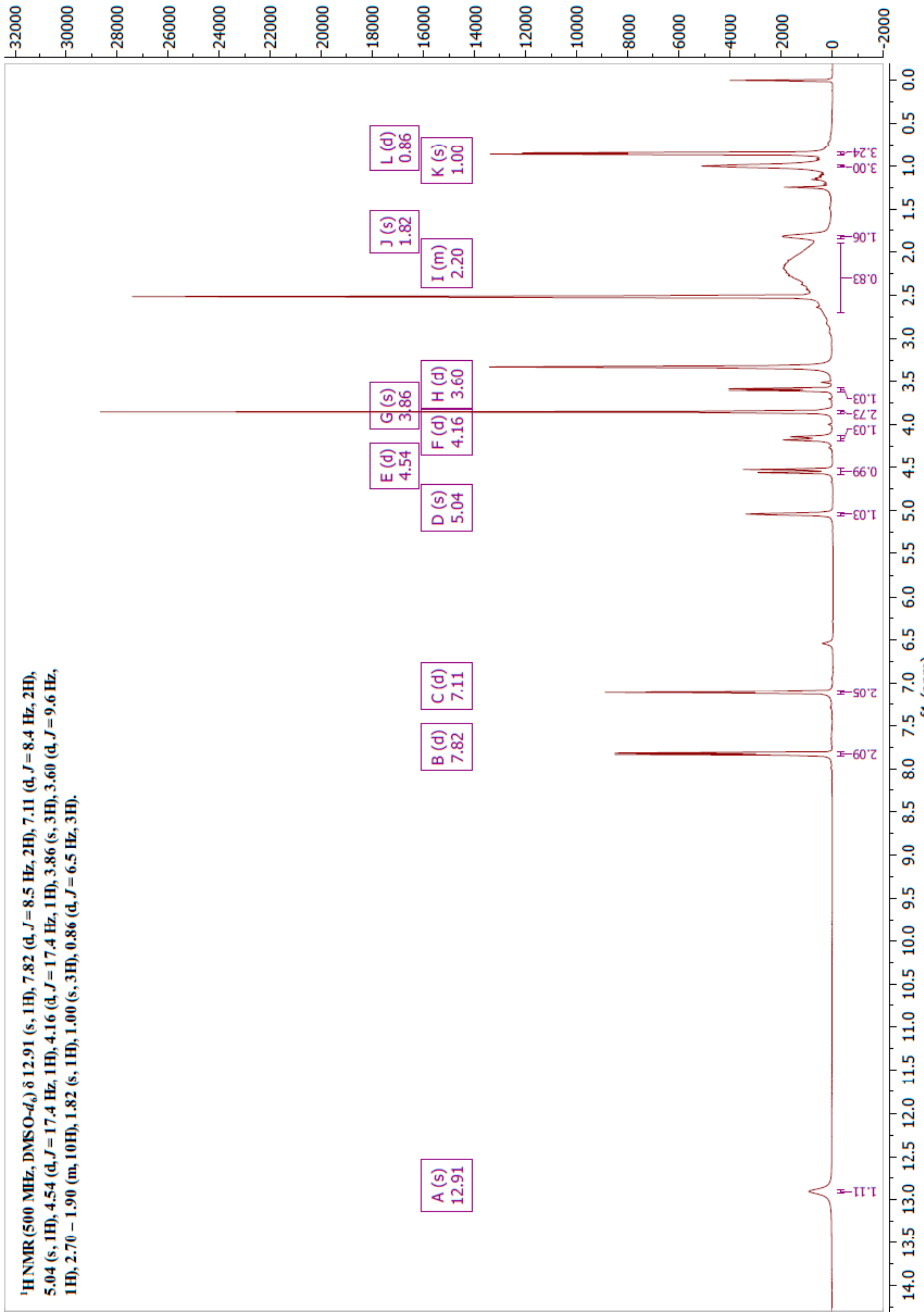

^{13}C NMR of Carboxylic Acid Closo-Carborane **9.**

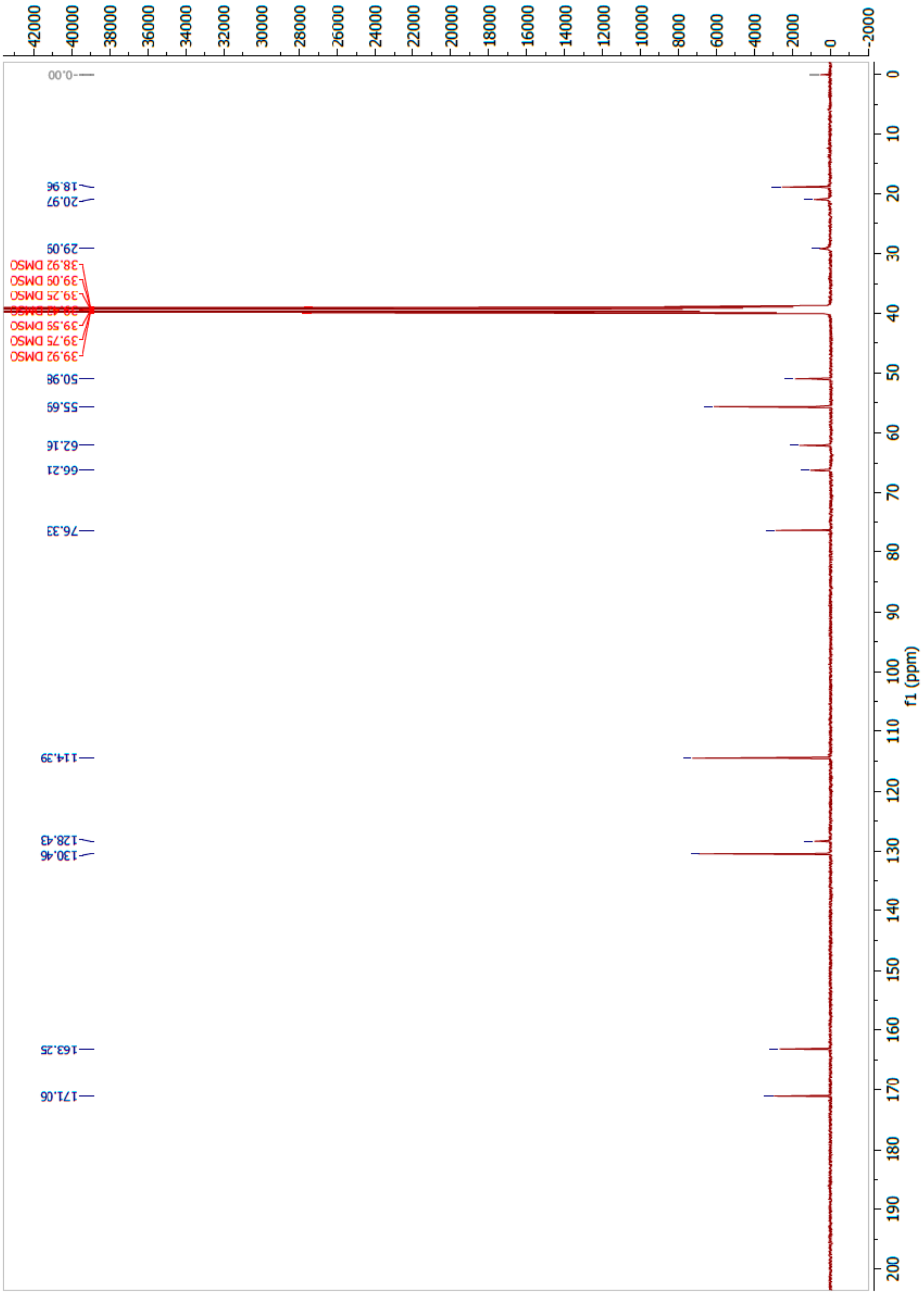

HRMS of Carboxylic Acid Closo-Carborane **9.**

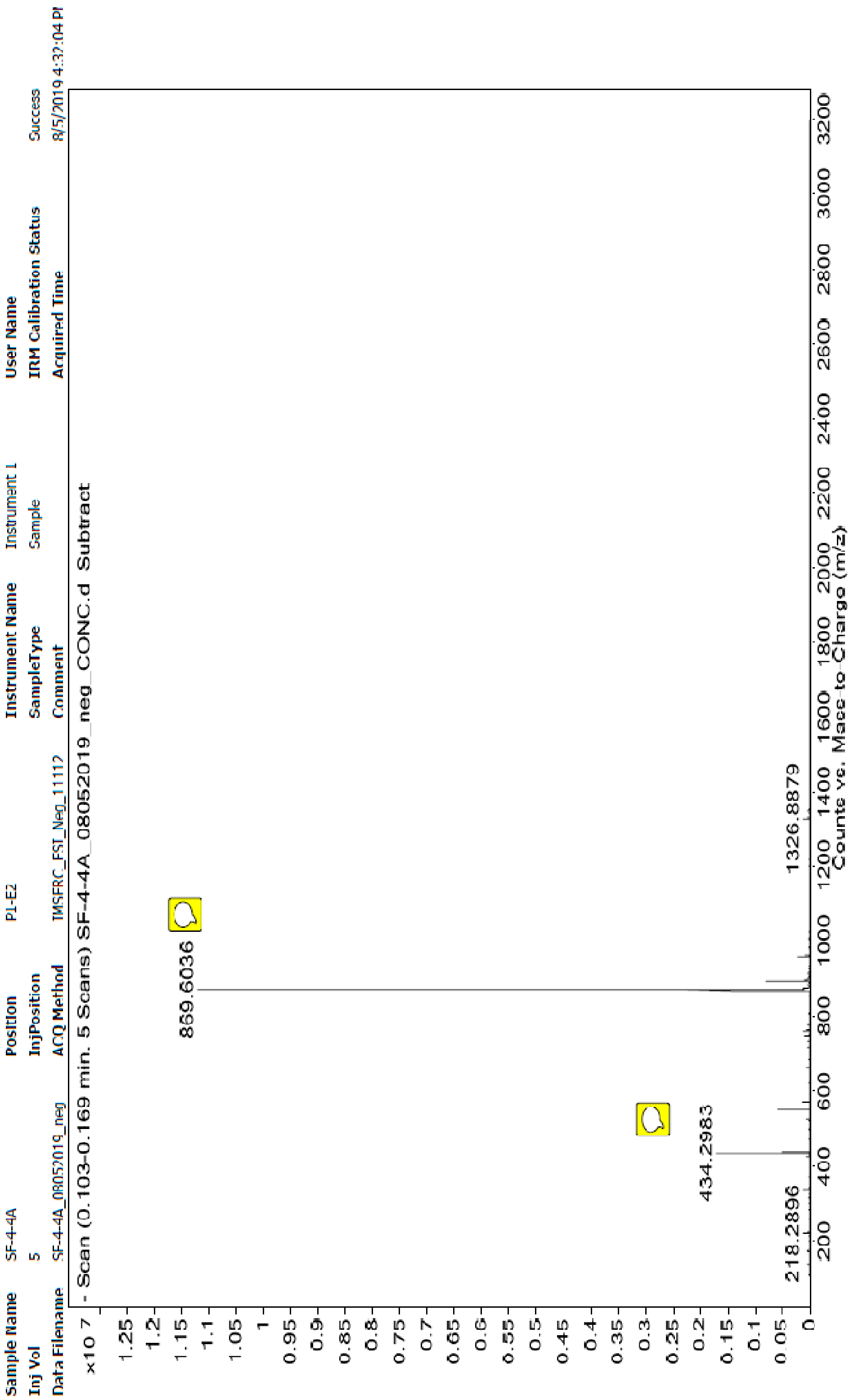

HPLC of THP-Protected Hydroxamate Closo-Carborane **10.**

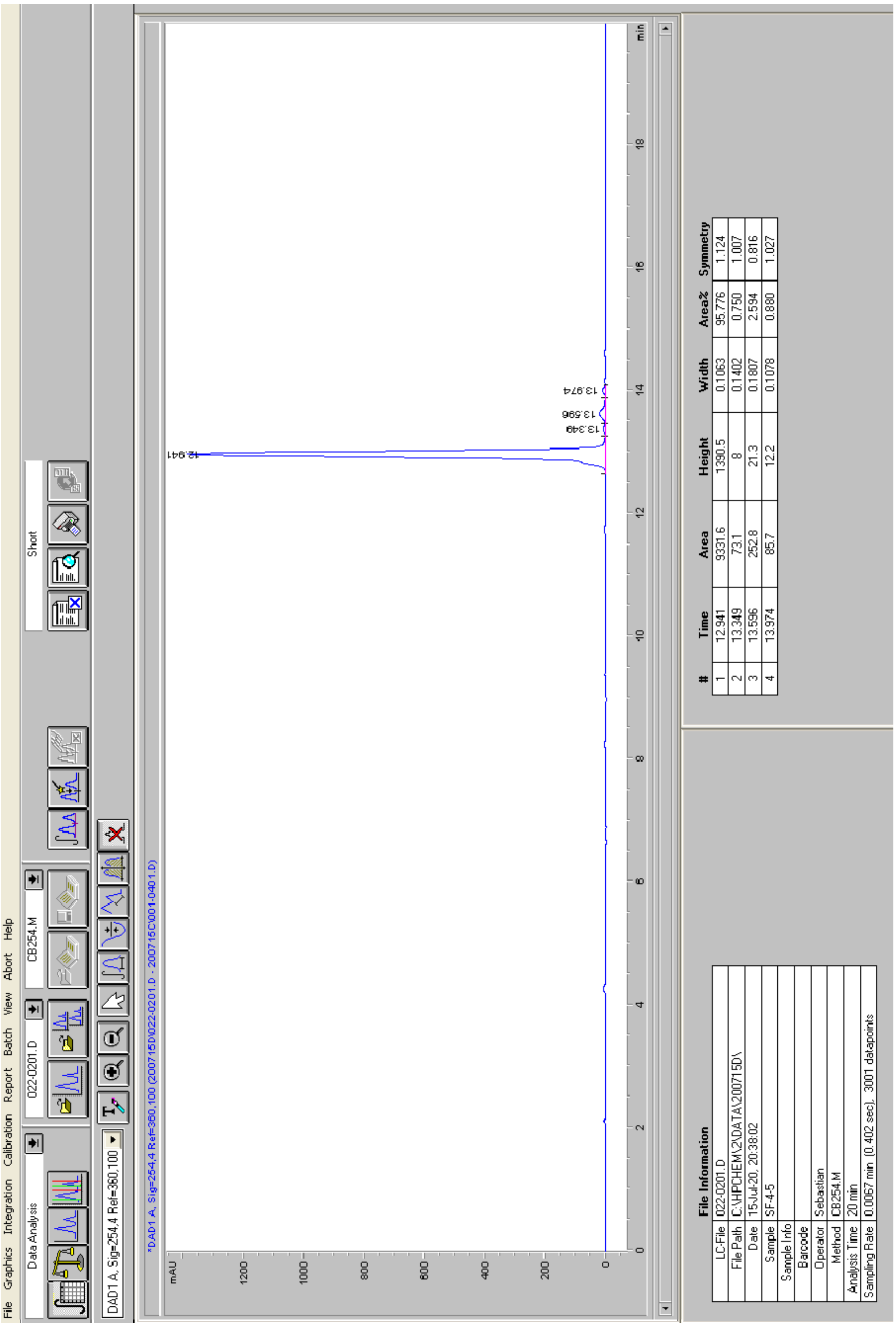

^{1}H NMR of THP-Protected Hydroxamate Closo-Carborane **10.**

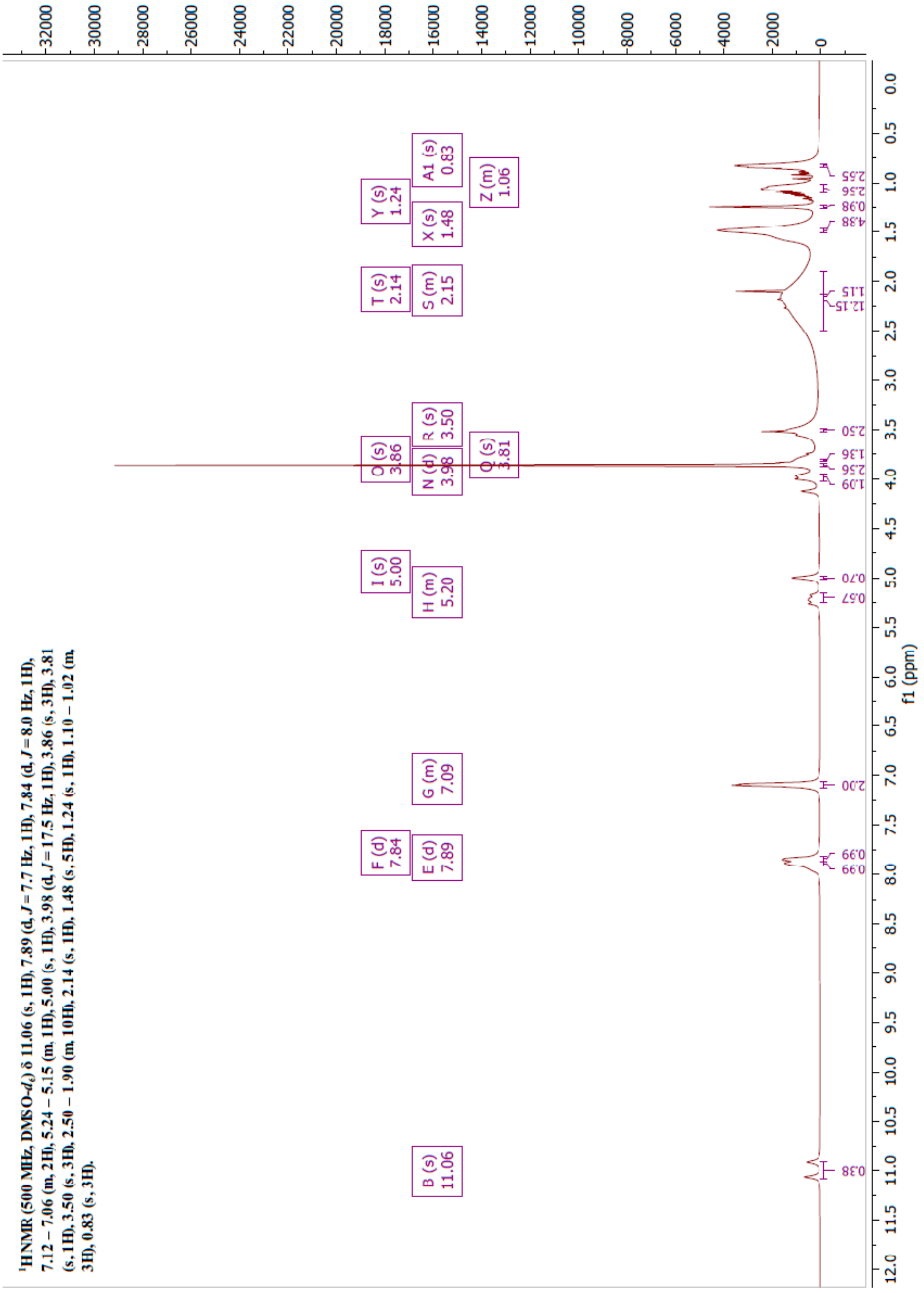

^{13}C NMR of THP-Protected Hydroxamate Closo-Carborane **10.**

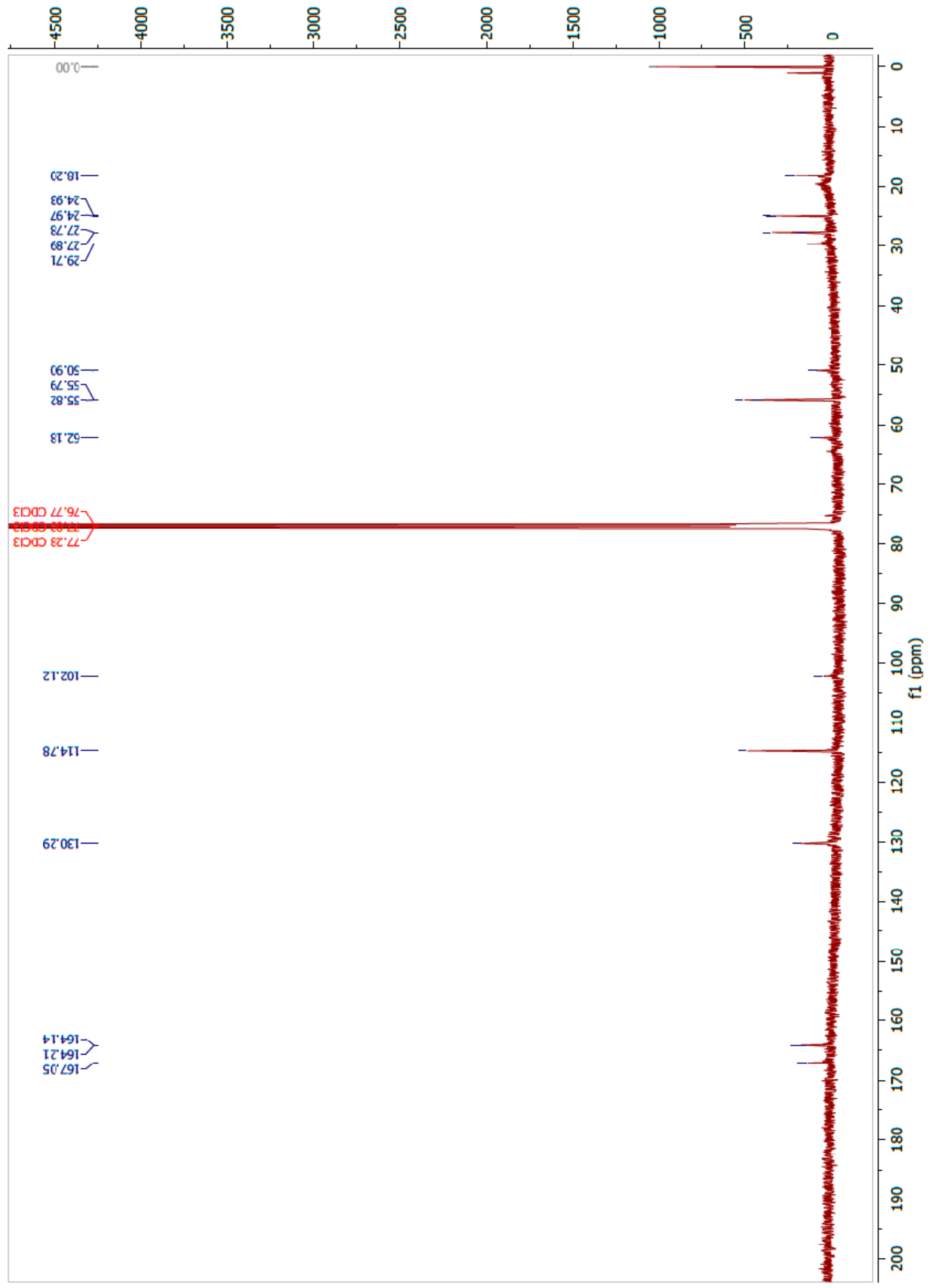

HRMS of THP-Protected Hydroxamate Closo-Carborane **10.**

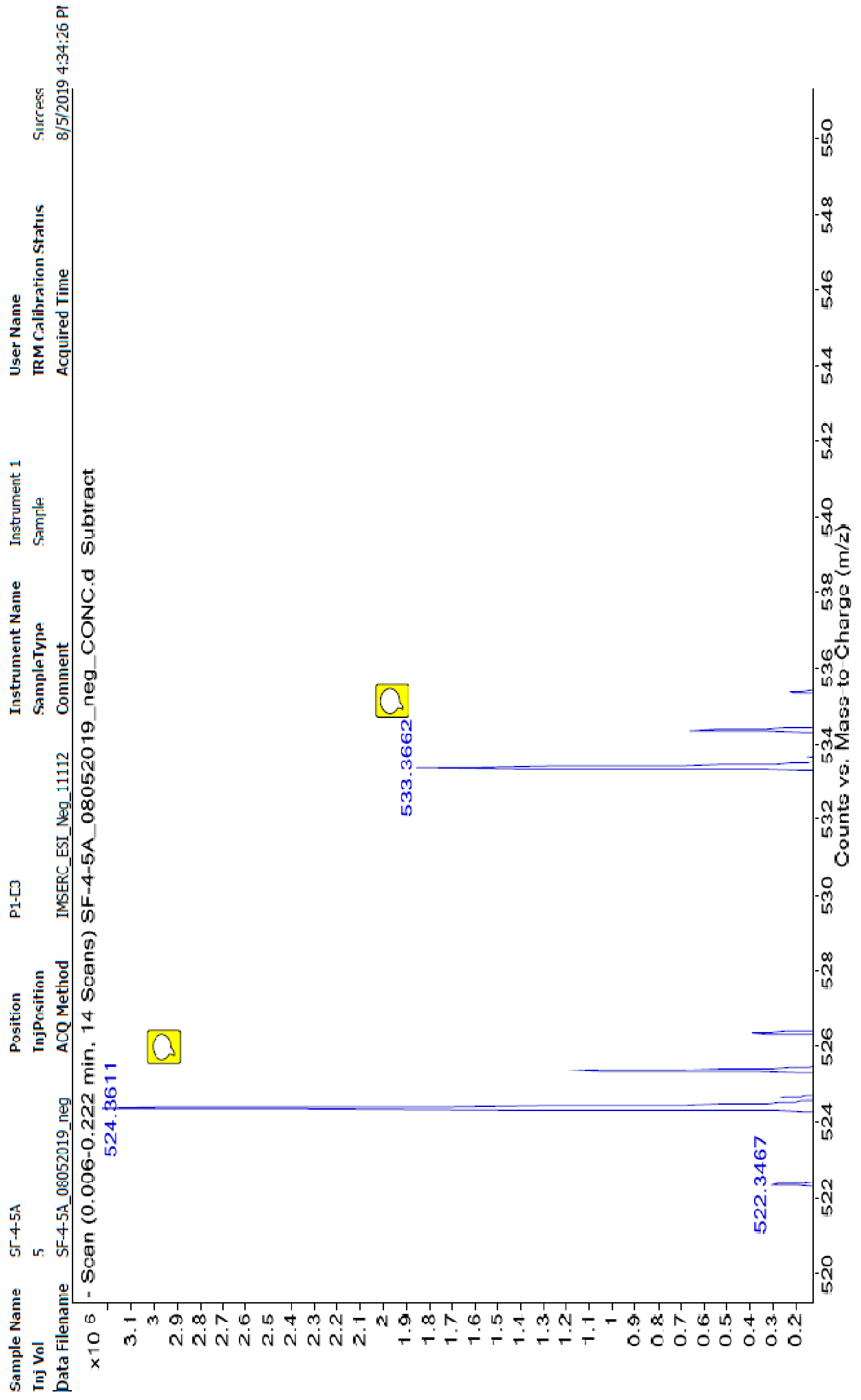

Sample Name	SF-4-5A	Position	P1-E3	Instrument Name	Instrument 1	User Name	
Inj Vol	5	InjPosition		SampleType	Sample	IRM Calibration Status	Success
Data Filename	SF-4-5A_08052019_neg	ACQ Method	IMSERC_ESI_Neg_11112	Comment		Acquired Time	8/5/2019 4:34:26 PI

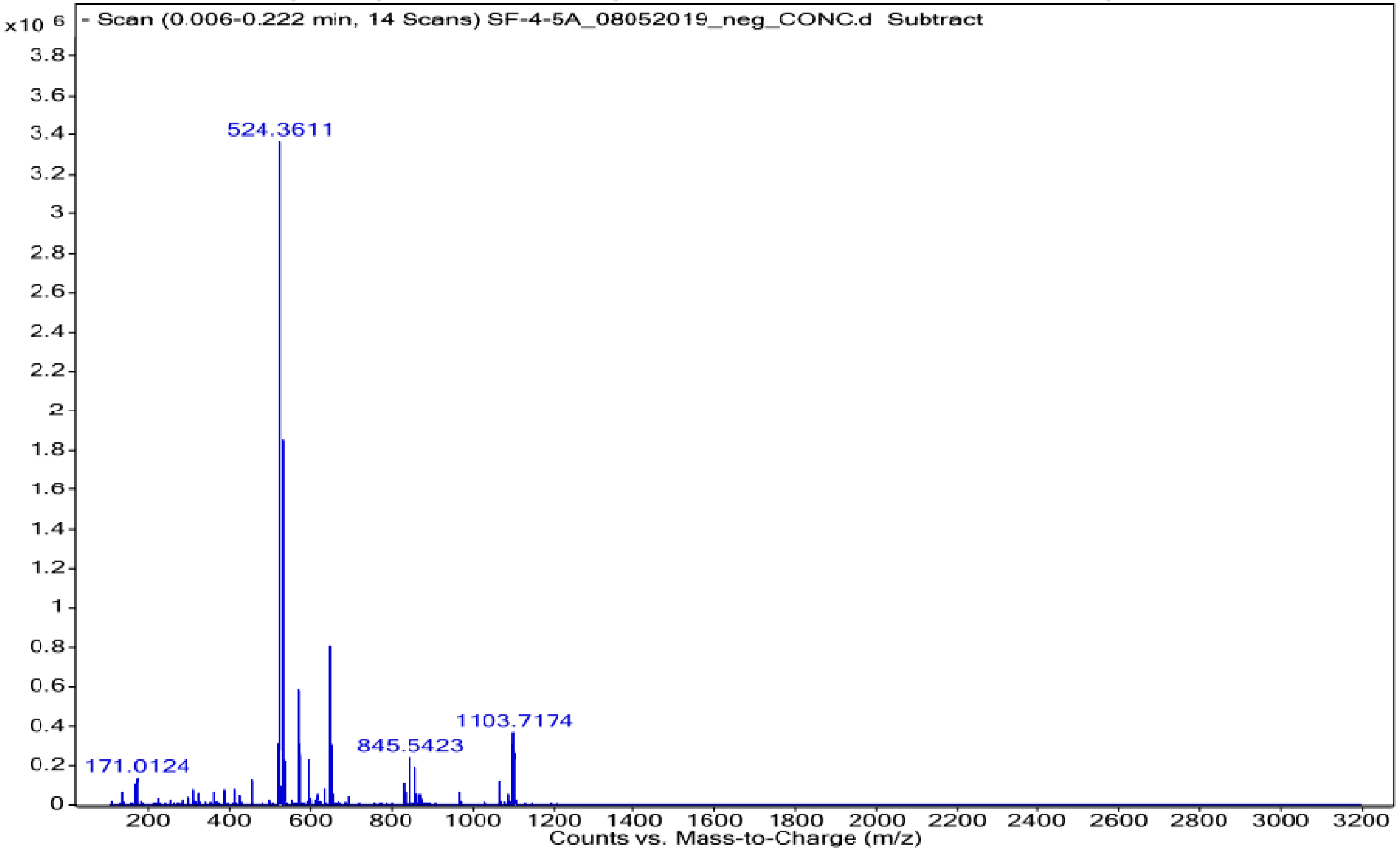

HPLC of Hydroxamic Acid Closo-Carborane **3.**

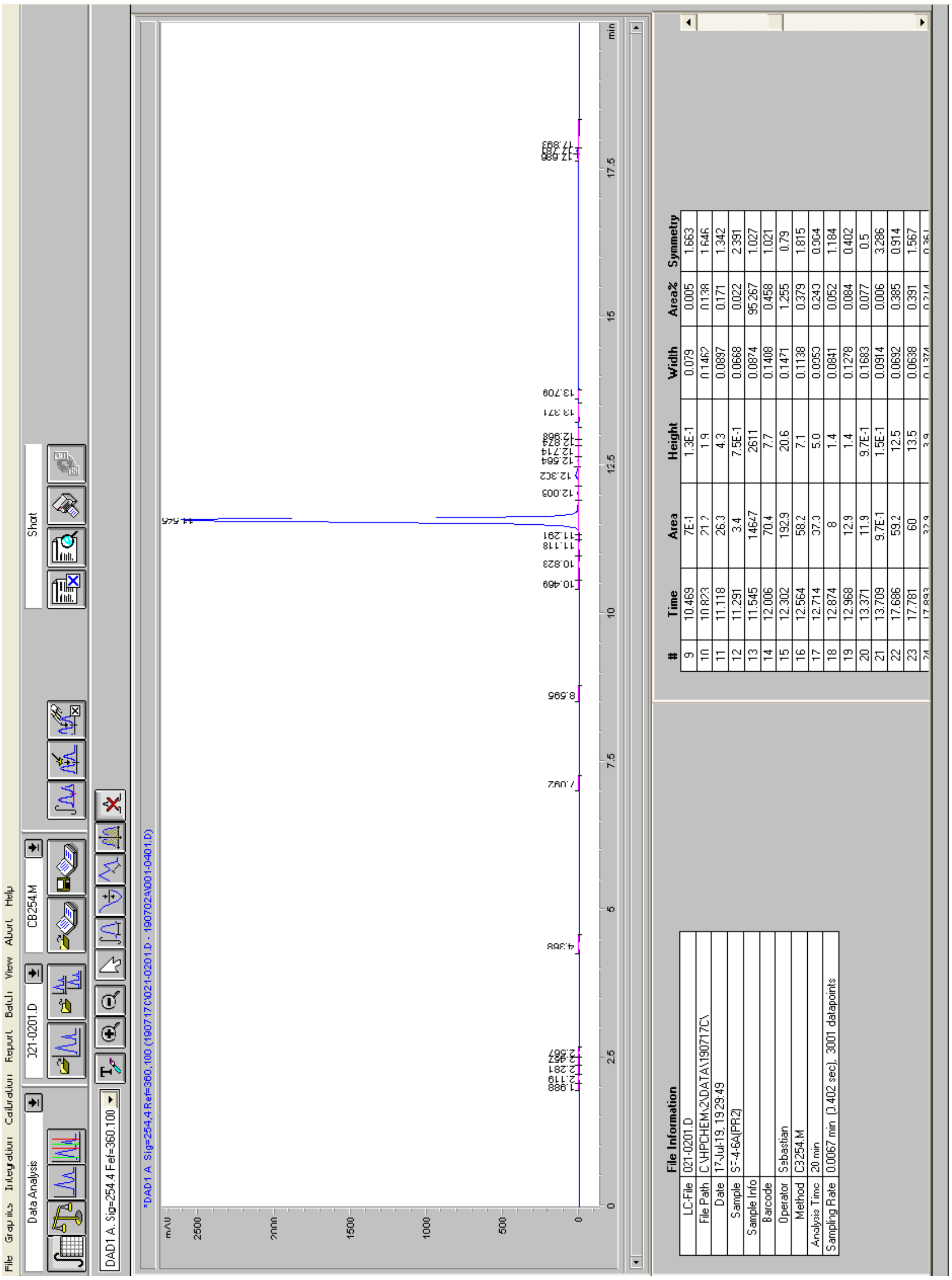

^{1}H NMR of Hydroxamic Acid Closo-Carborane **3.**

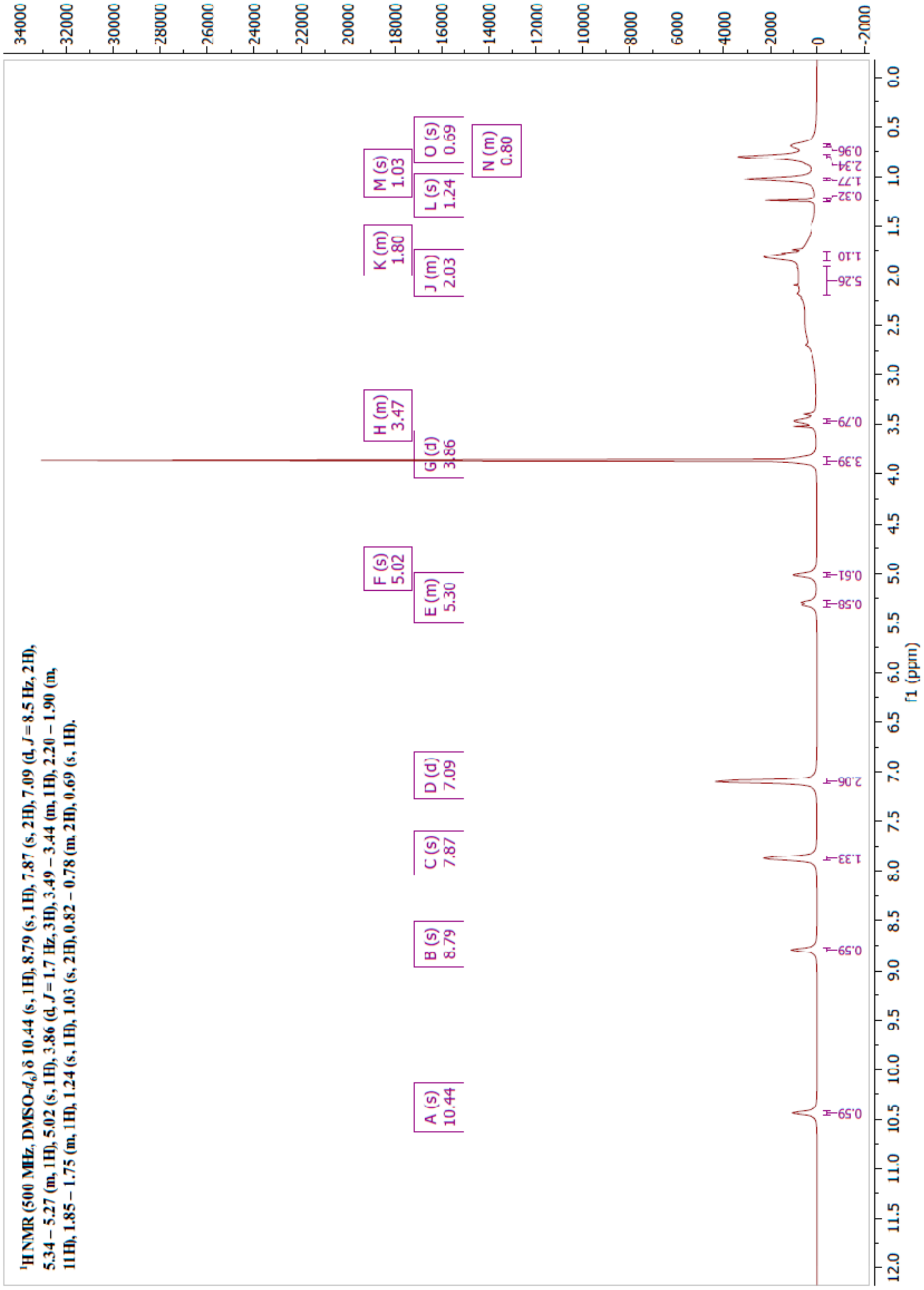

^{13}C NMR of Hydroxamic Acid Closo-Carborane **3.**

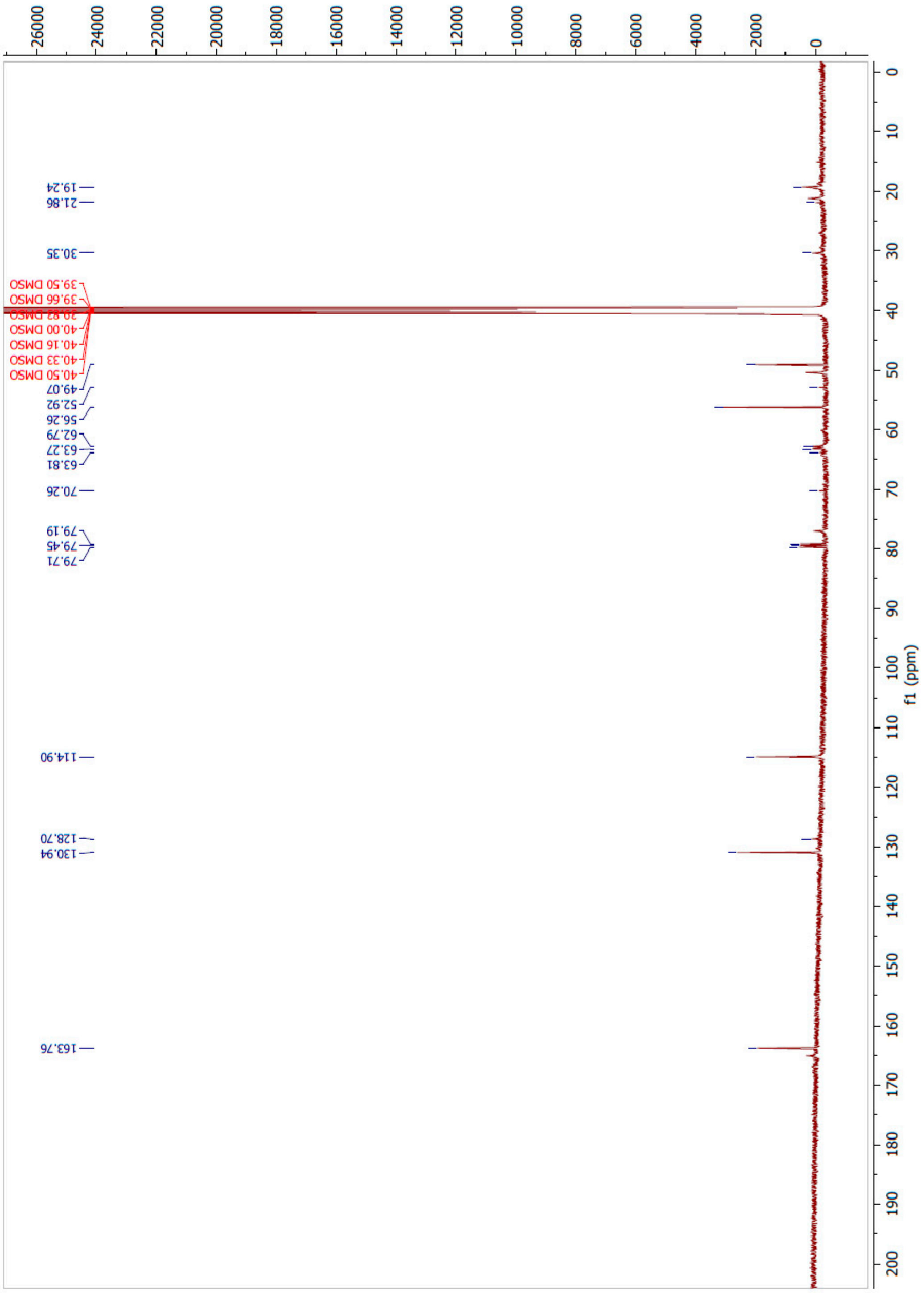

HRMS of Hydroxamic Acid Closo-Carborane **3.**

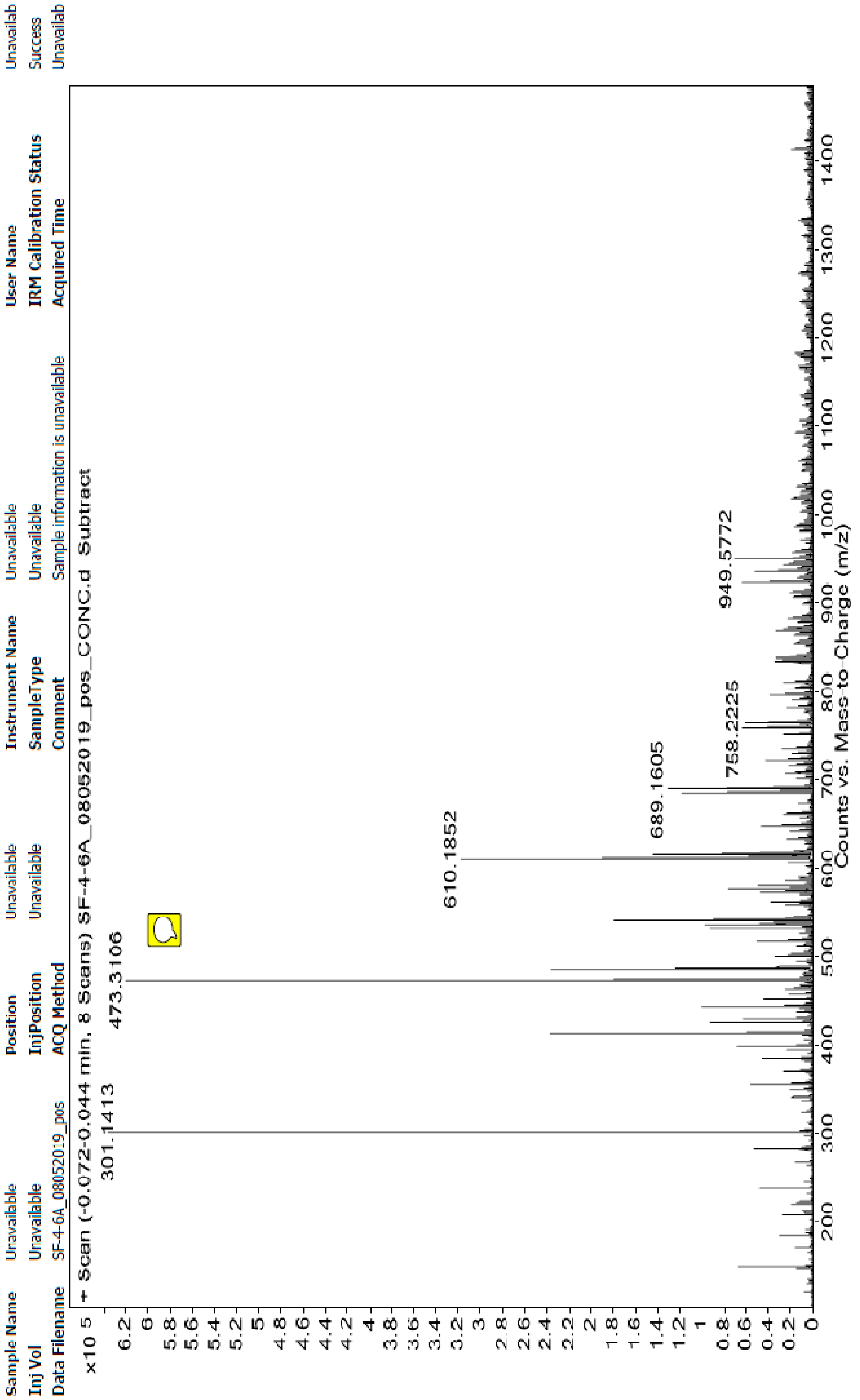

**Sample Name**	Unavailable	**Position**	Unavailable	**Instrument Name**	Unavailable	**User Name**	Unavailab
**Inj Vol**	Unavailable	**InjPosition**	Unavailable	**SampleType**	Unavailable	**IRM Calibration Status**	Success
**Data Filename**	SF-4-6A_08052019_pos	**ACQ Method**		**Comment**	Sample information is unavailable	**Acquired Time**	Unavailab

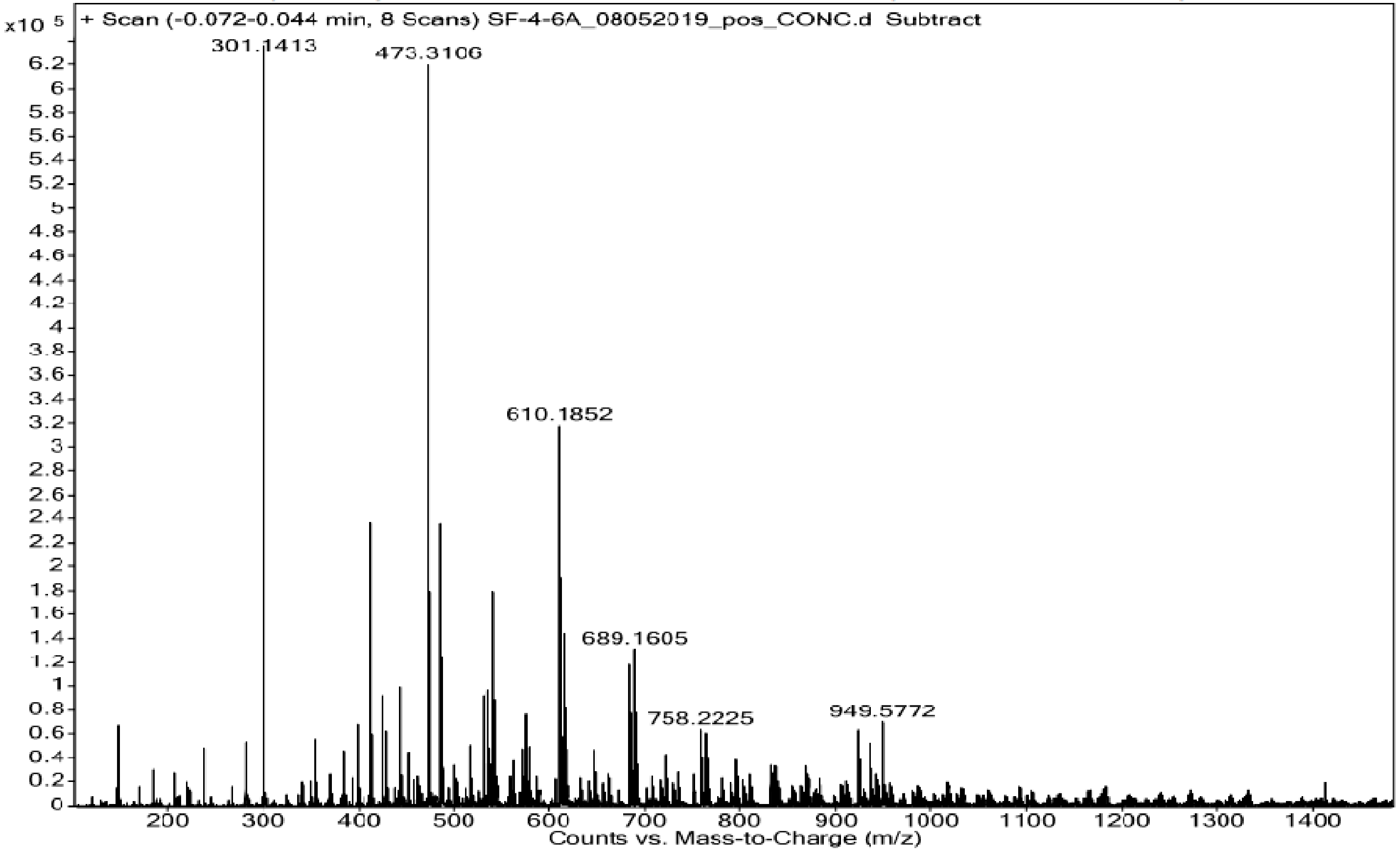

HPLC of Methyl (*R*)-2-((4-methoxy-*N*-(pyridine-3-ylmethyl) phenyl) sulfonamido) pent-4-ynoate **(14).**

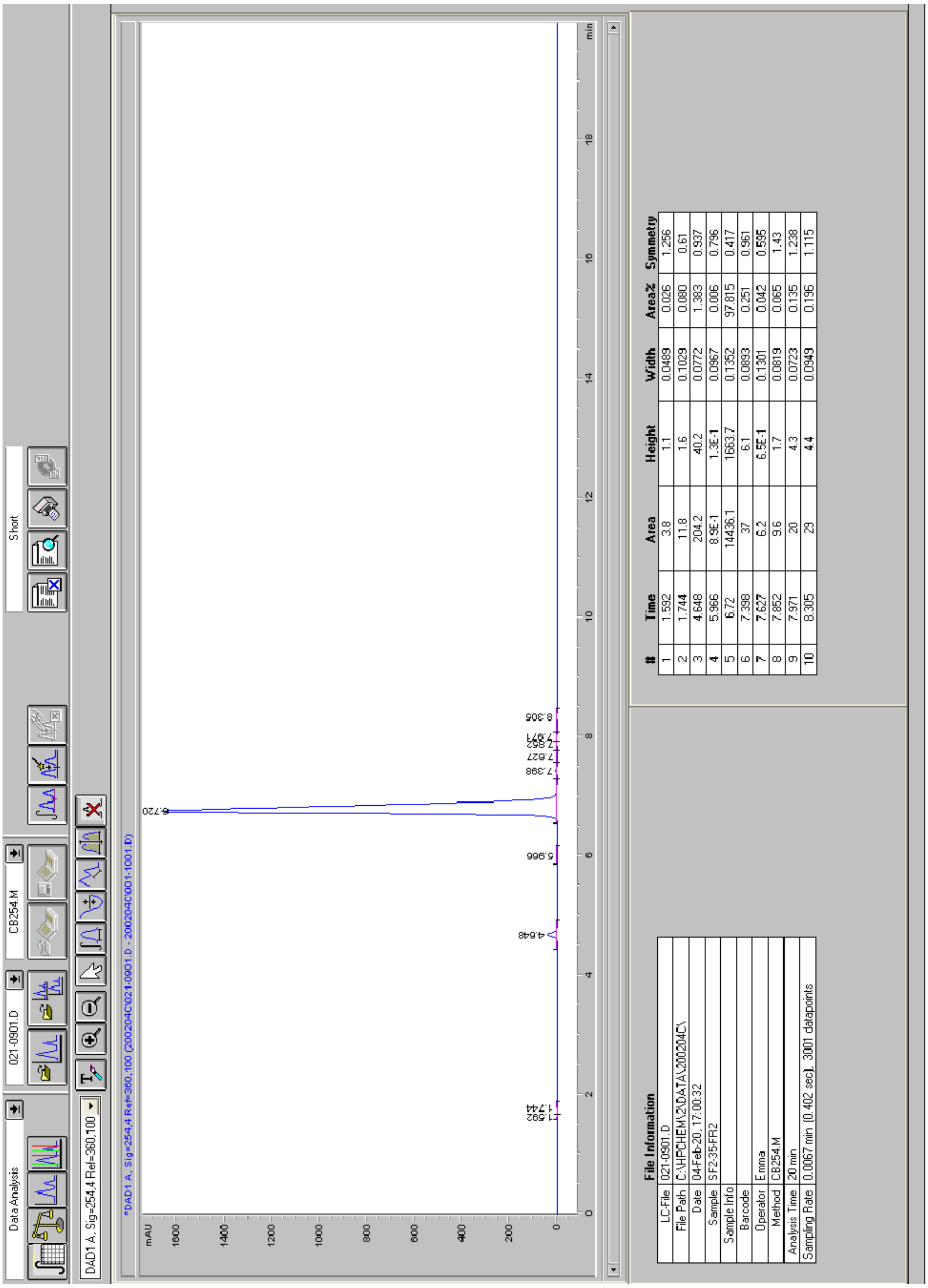

#	Time	Area	Height	Width	Area%	Symmetry
1	1.592	3.8	1.1	0.0489	0.026	1.256
2	1.744	11.8	1.6	0.1029	0.080	0.61
3	4.648	204.2	40.2	0.0772	1.383	0.937
4	5.966	8.9E-1	1.3E-1	0.0967	0.006	0.796
5	6.72	14436.1	1663.7	0.1352	97.815	0.417
6	7.398	37	6.1	0.0893	0.251	0.961
7	7.627	6.2	6.5E-1	0.1301	0.042	0.595
8	7.852	9.6	1.7	0.0819	0.065	1.43
9	7.971	20	4.3	0.0723	0.135	1.238
10	8.305	29	4.4	0.0949	0.196	1.115

LC-File	021-0901.D
File Path	C:\HPCHEM\2\DATA\200204C\
Date	04-Feb-20, 17:00:32
Sample	SF2-35-FR2
Sample Info	
Barcode	
Operator	Emma
Method	CB254.M
Analysis Time	20 min
Sampling Rate	0.0067 min (0.402 sec), 3001 datapoints

^{1}H NMR of Methyl (*R*)-2-((4-methoxy-*N*-(pyridine-3-ylmethyl) phenyl) sulfonamido) pent-4-ynoate **(14).**

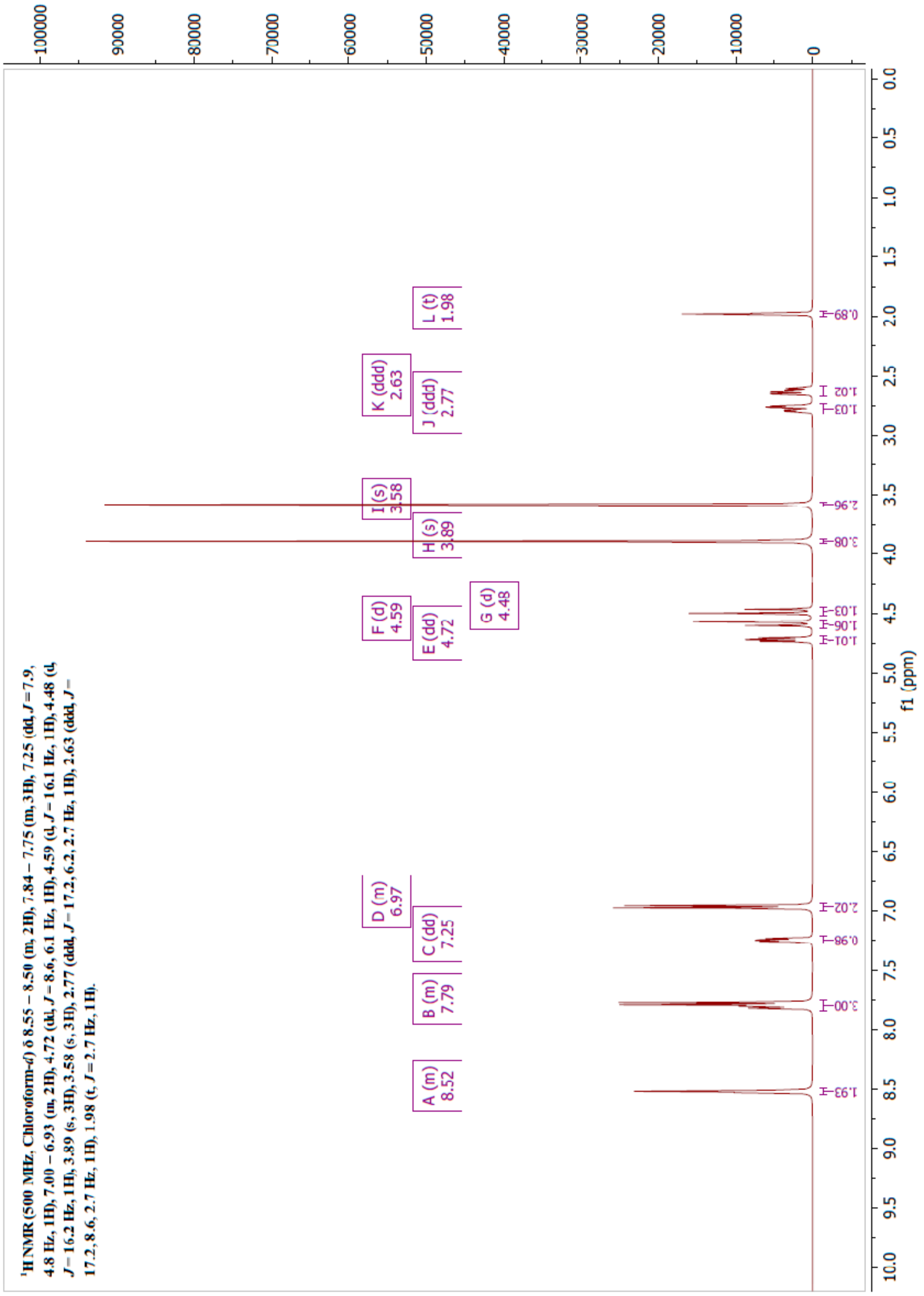

^{13}C NMR of Methyl (*R*)-2-((4-methoxy-*N*-(pyridine-3-ylmethyl) phenyl) sulfonamido) pent-4-ynoate

(14).

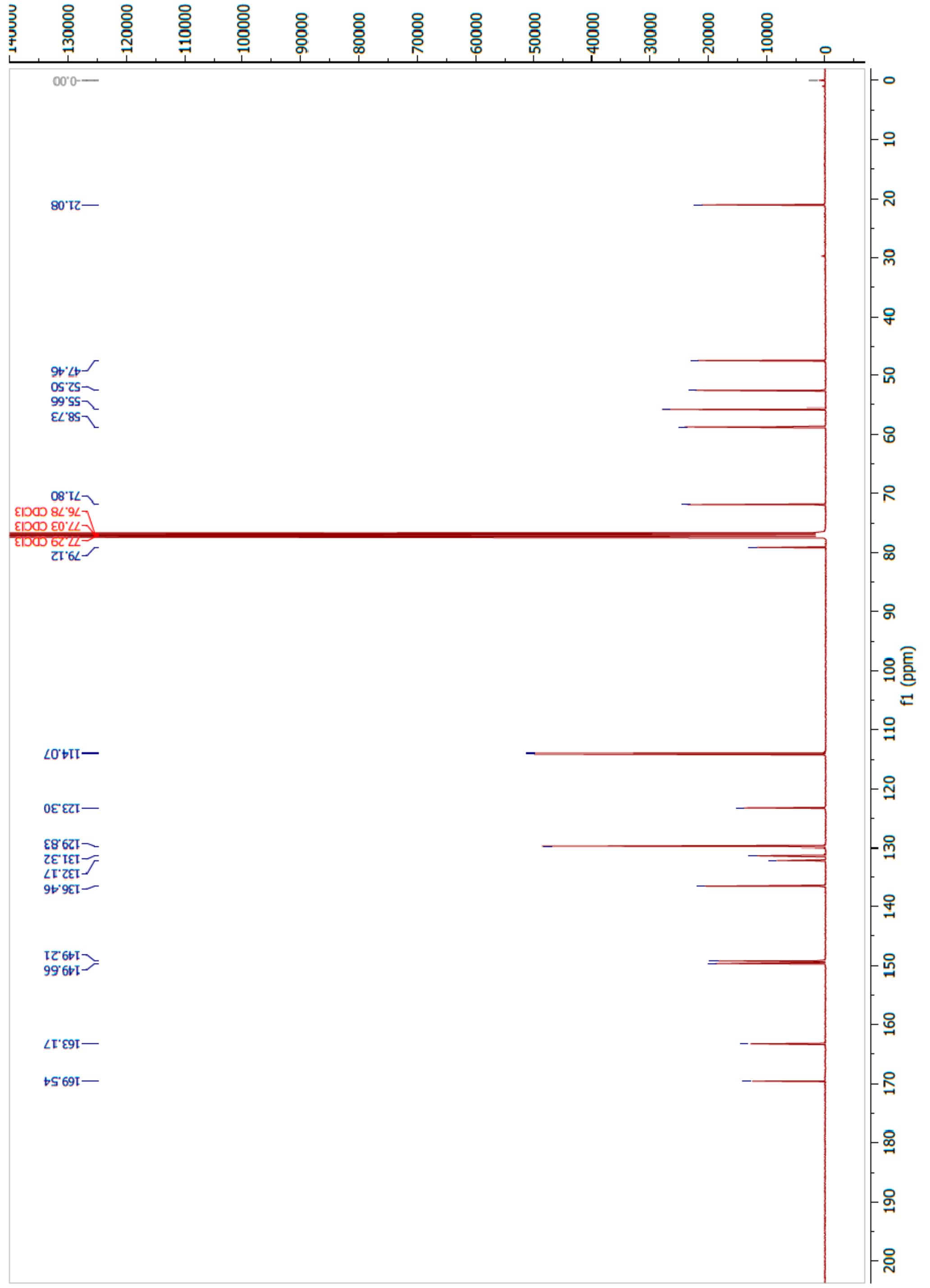

HRMS of Methyl (*R*)-2-((4-methoxy-*N*-(pyridine-3-ylmethyl) phenyl) sulfonamido) pent-4-ynoate **(14).**

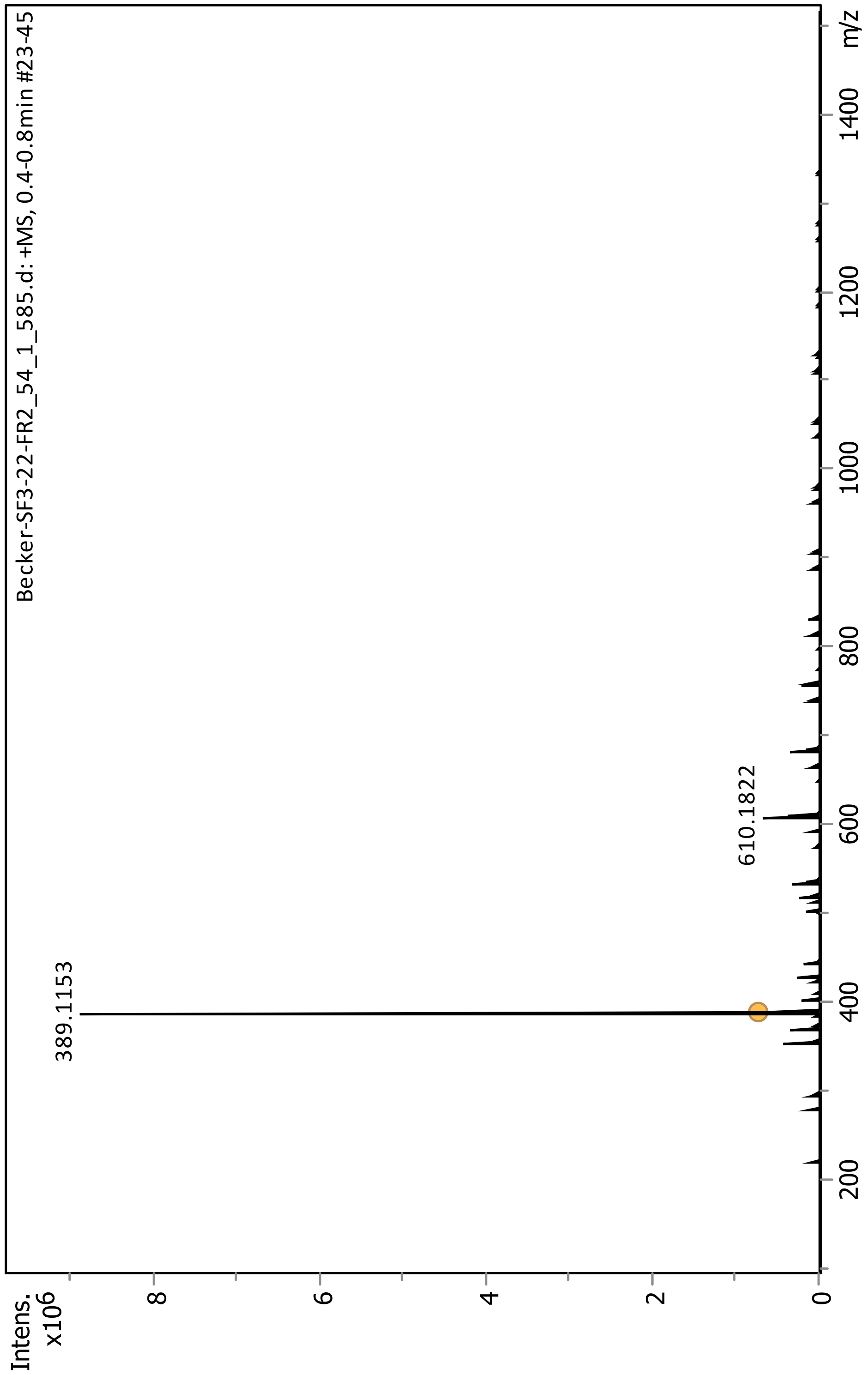

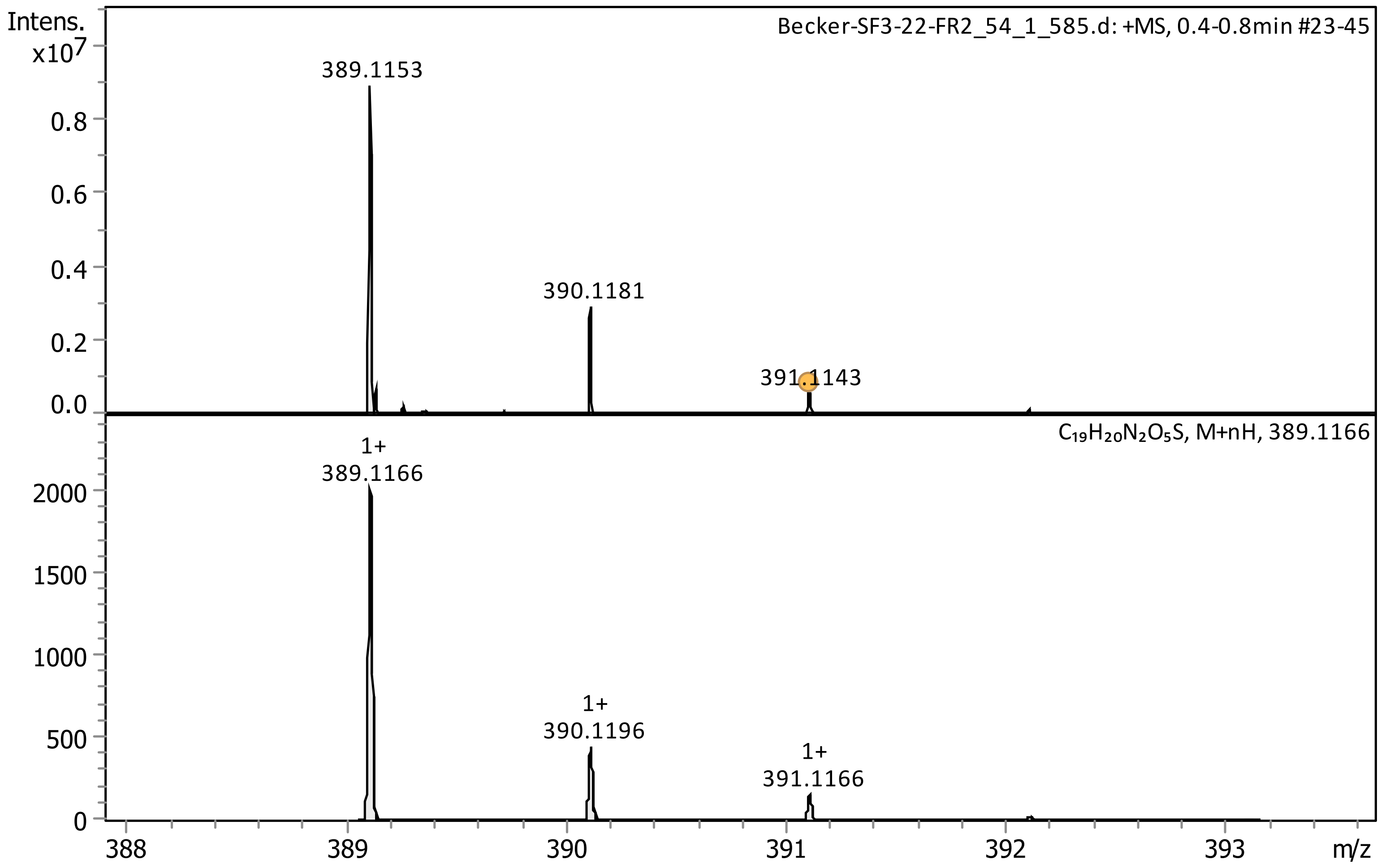

Intens.
x10^7
0.8
0.6
0.4
0.2
0.0
Becker-SF3-22-FR2_54_1_585.d: +MS, 0.4-0.8min #23-45
389.1153
390.1181
391.1143
C19H20N2O5S, M+nH, 389.1166
1+
389.1166
1+
390.1196
1+
391.1166
2000
1500
1000
500
0
388
389
390
391
392
393
m/z

HPLC of (R)-2-((4-methoxy-N-(pyridin-3-ylmethyl)phenyl)sulfonamido)pent-4-ynoic acid **(15).**

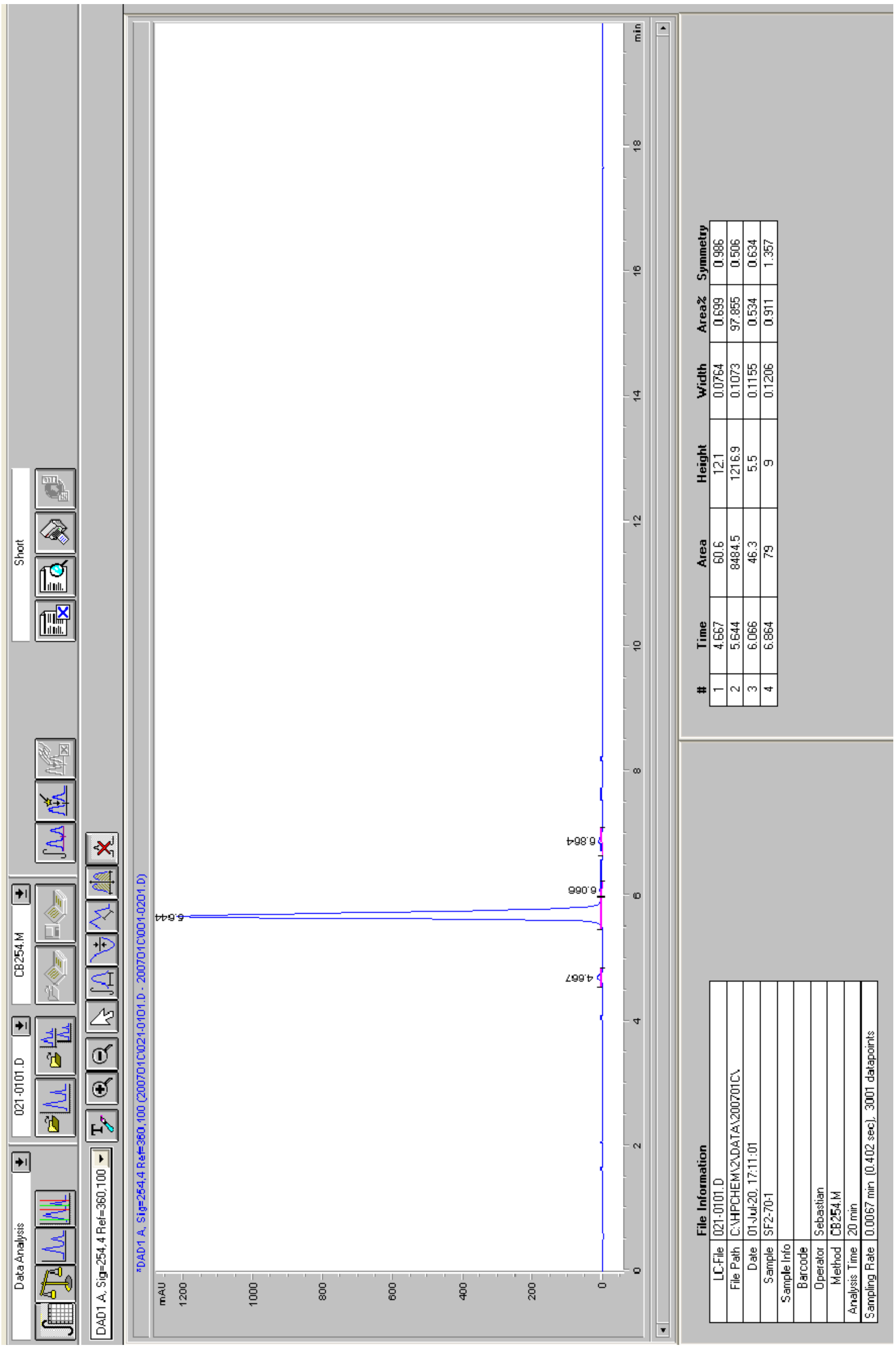

#	Time	Area	Height	Width	Area%	Symmetry
1	4.667	60.6	12.1	0.0764	0.699	0.986
2	5.644	8484.5	1216.9	0.1073	97.855	0.506
3	6.066	46.3	5.5	0.1155	0.534	0.634
4	6.864	79	9	0.1206	0.911	1.357

File Information

LC-File	021-0101.D
File Path	C:\HPCHEM\2\DATA\200701C\
Date	01-Jul-20, 17:11:01
Sample	SF2-70-1
Sample Info	
Barcode	
Operator	Sebastian
Method	CB254.M
Analysis Time	20 min
Sampling Rate	0.0067 min (0.402 sec), 3001 datapoints

^{1}H NMR of (R)-2-((4-methoxy-N-(pyridin-3-ylmethyl)phenyl)sulfonamido)pent-4-ynoic acid **(15).**

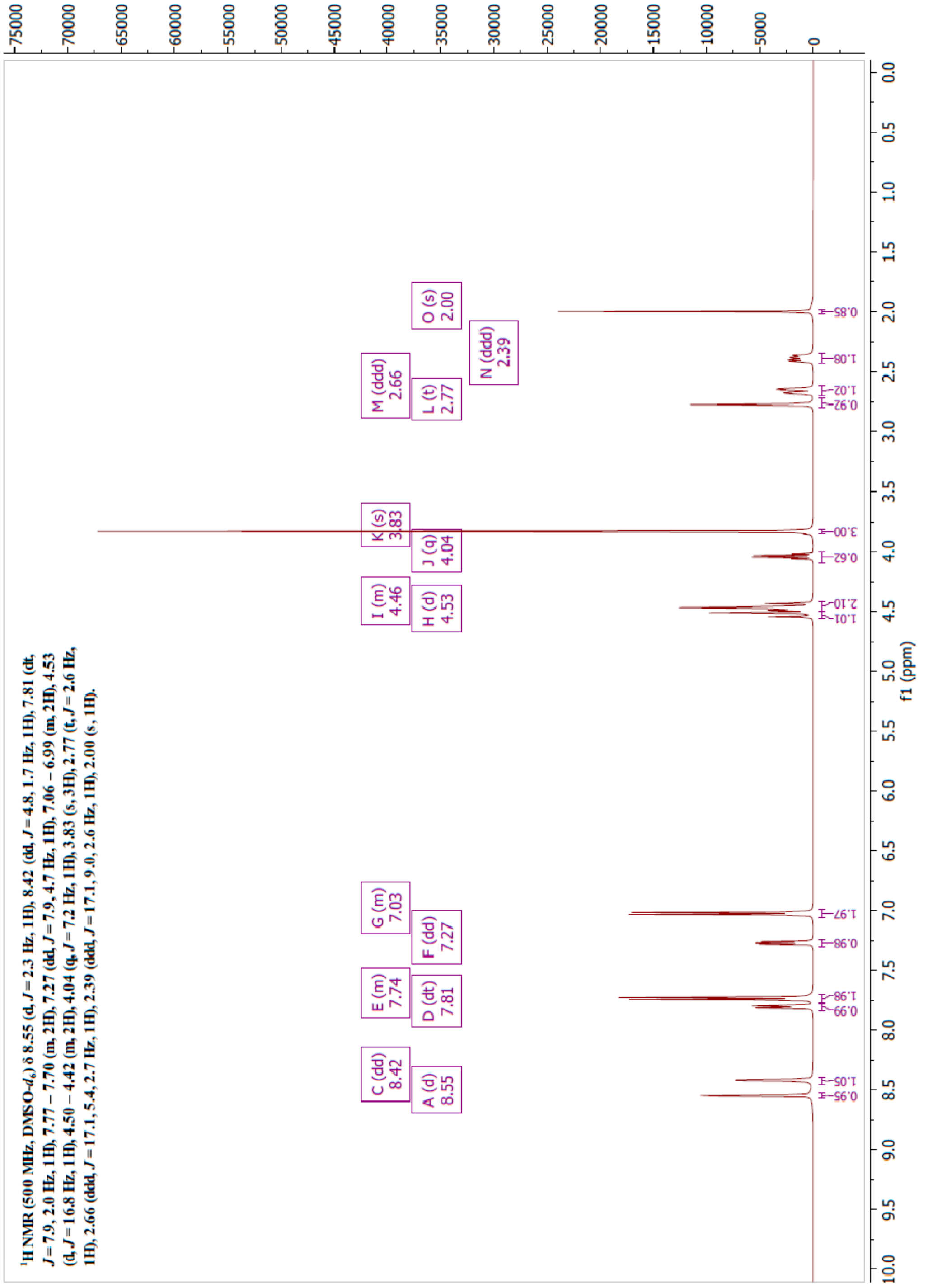

^{13}C NMR of (R)-2-((4-methoxy-N-(pyridin-3-ylmethyl)phenyl)sulfonamido)pent-4-ynoic acid **(15).**

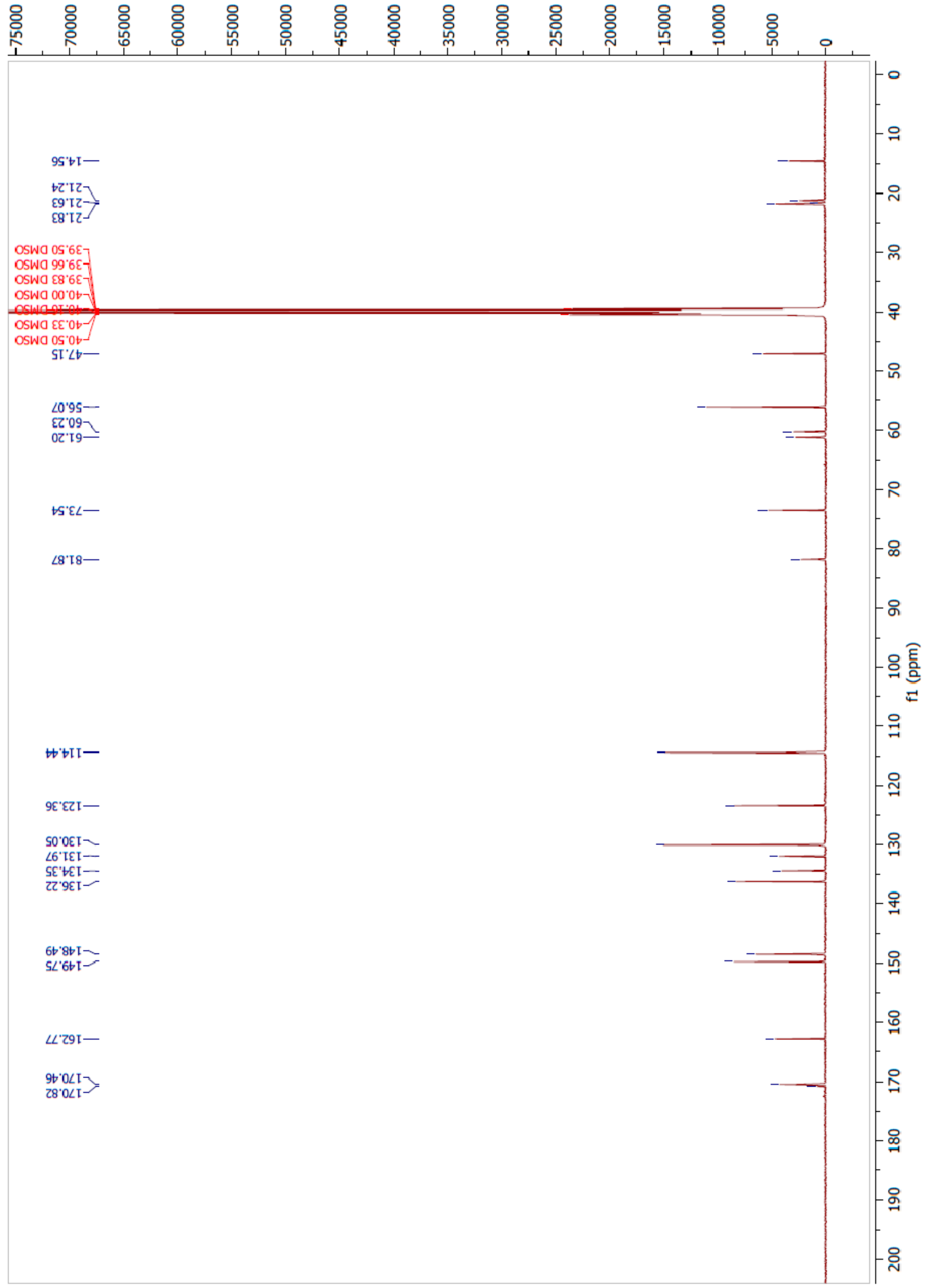

HRMS of (R)-2-((4-methoxy-N-(pyridin-3-ylmethyl)phenyl)sulfonamido)pent-4-ynoic acid **(15).**

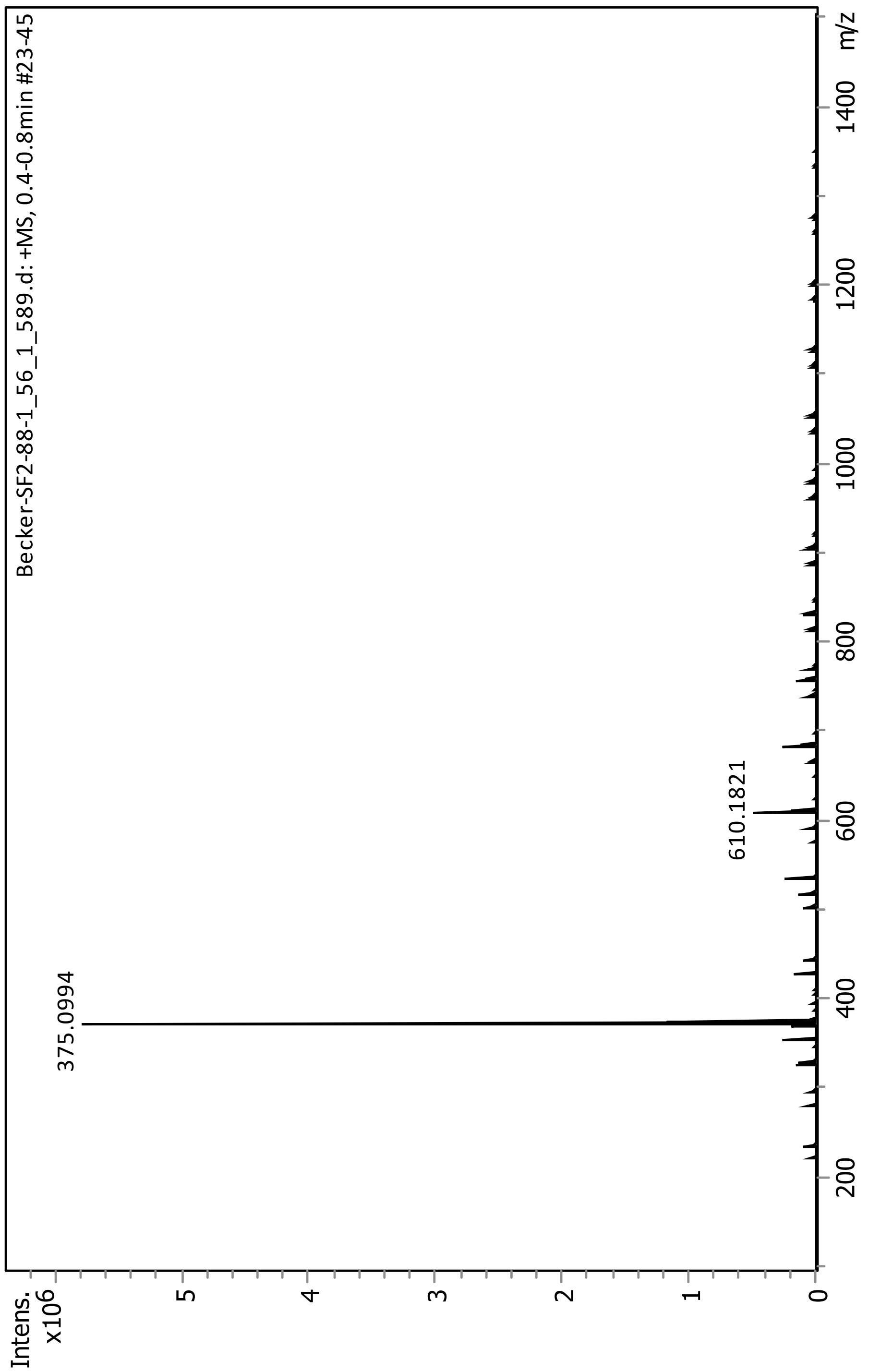

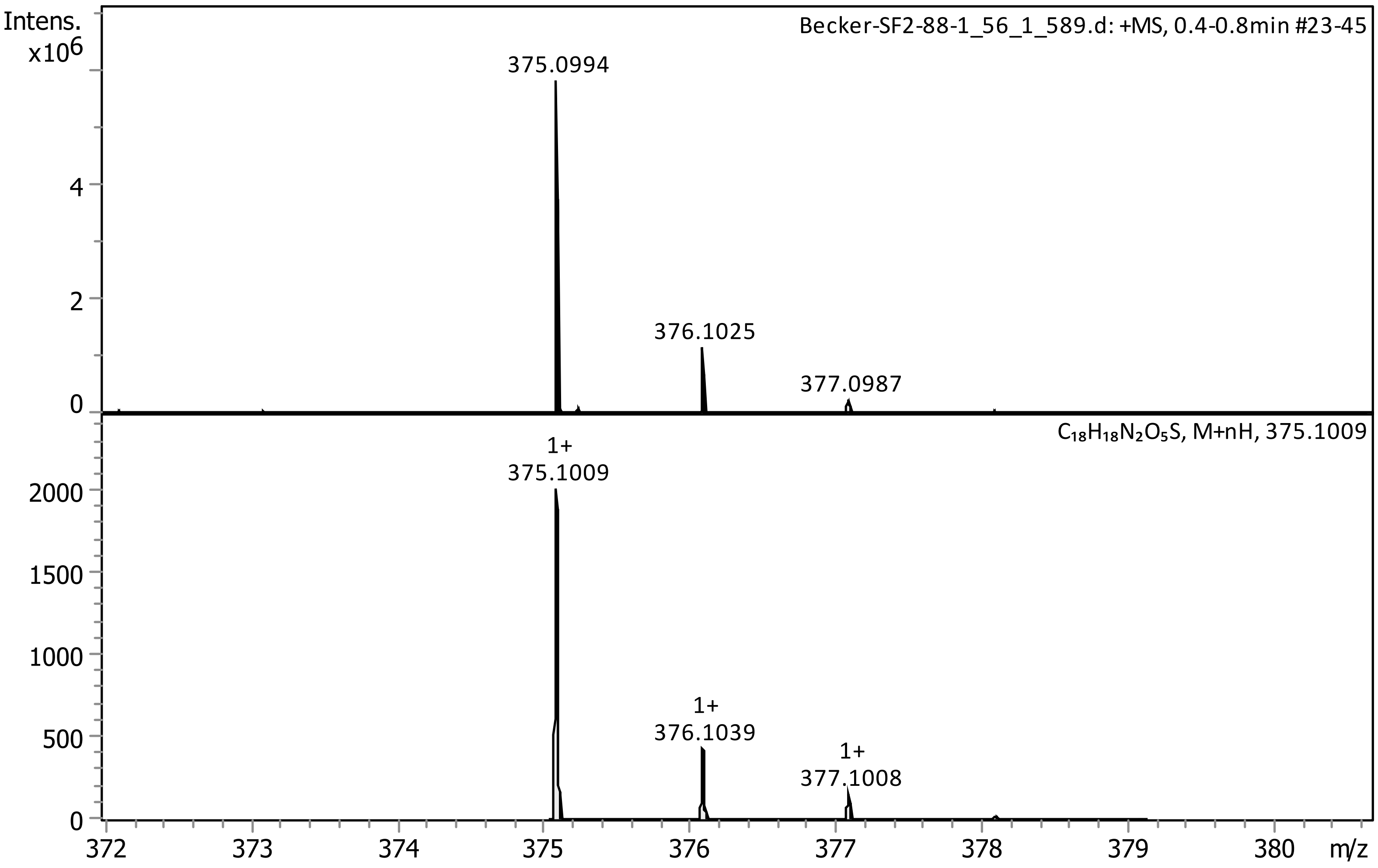

Intens.
x10^6
Becker-SF2-88-1_56_1_589.d: +MS, 0.4-0.8min #23-45
375.0994
376.1025
377.0987
C18H18N2O5S, M+nH, 375.1009
1+
375.1009
1+
376.1039
1+
377.1008
m/z

HPLC of 4-methoxy-N-((2R)-1-oxo-1-(((tetrahydro-2H-pyran-2-yl)oxy)-l2-azaneyl)pent-4-yn-2-yl)-N-(pyridin-3-ylmethyl)benzenesulfonamide **(16).**

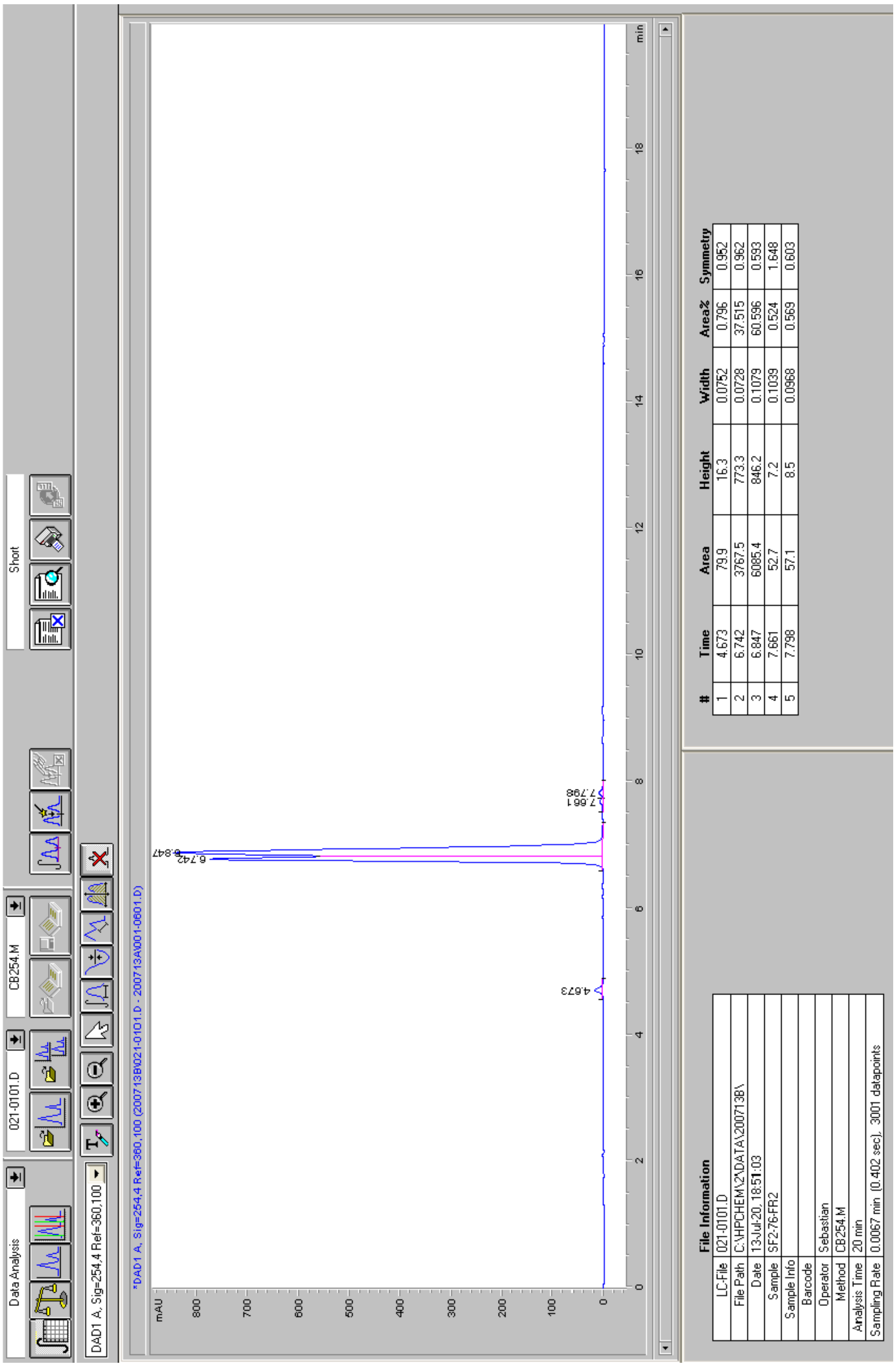

^{1}H NMR of 4-methoxy-N-((2R)-1-oxo-1-(((tetrahydro-2H-pyran-2-yl)oxy)-l2-azaneyl)pent-4-yn-2-yl)-N-(pyridin-3-ylmethyl)benzenesulfonamide **(16).**

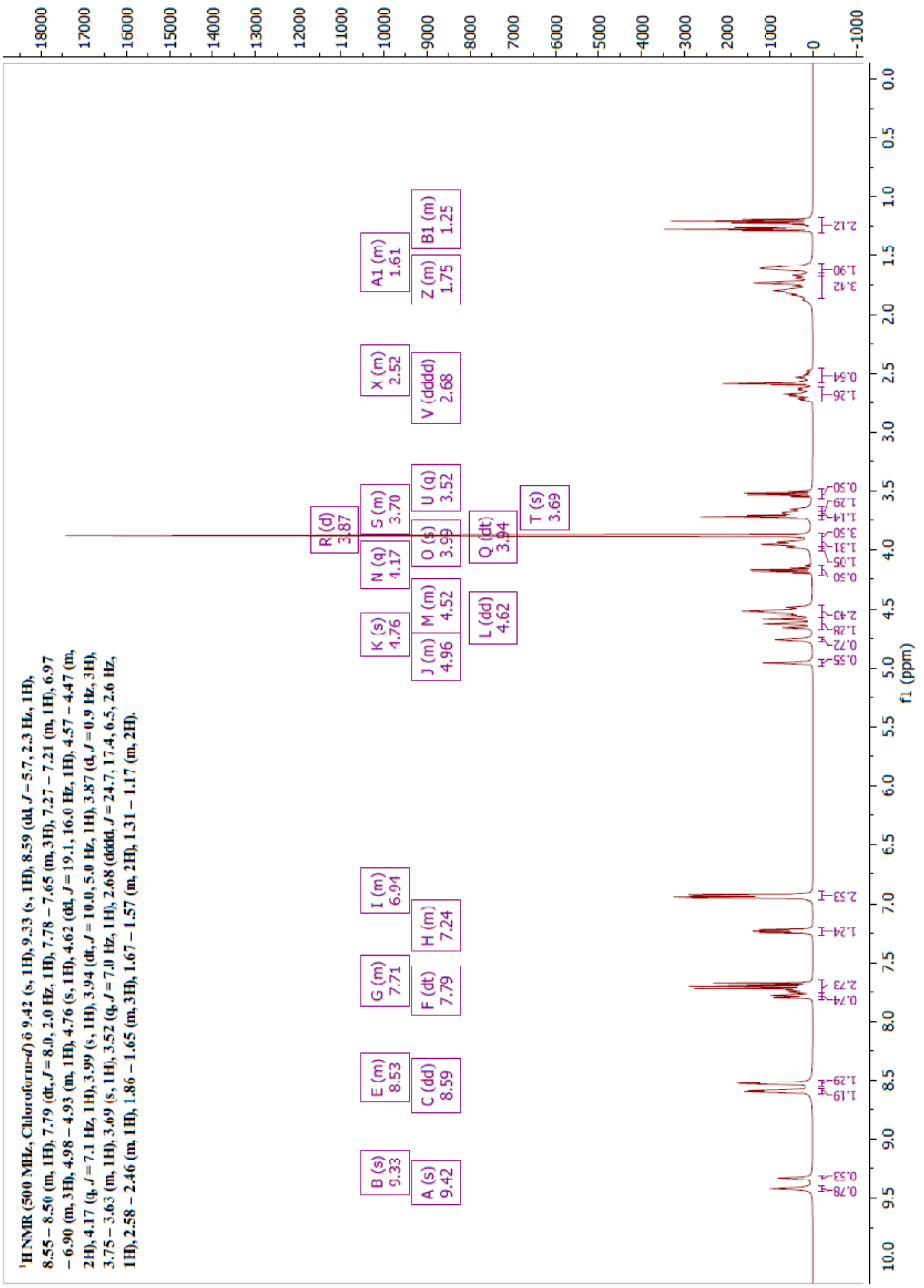

13C NMR of 4-methoxy-N-((2R)-1-oxo-1-(((tetrahydro-2H-pyran-2-yl)oxy)-l2-azaneyl)pent-4-yn-2-yl)-N-(pyridin-3-ylmethyl)benzenesulfonamide **(16).**

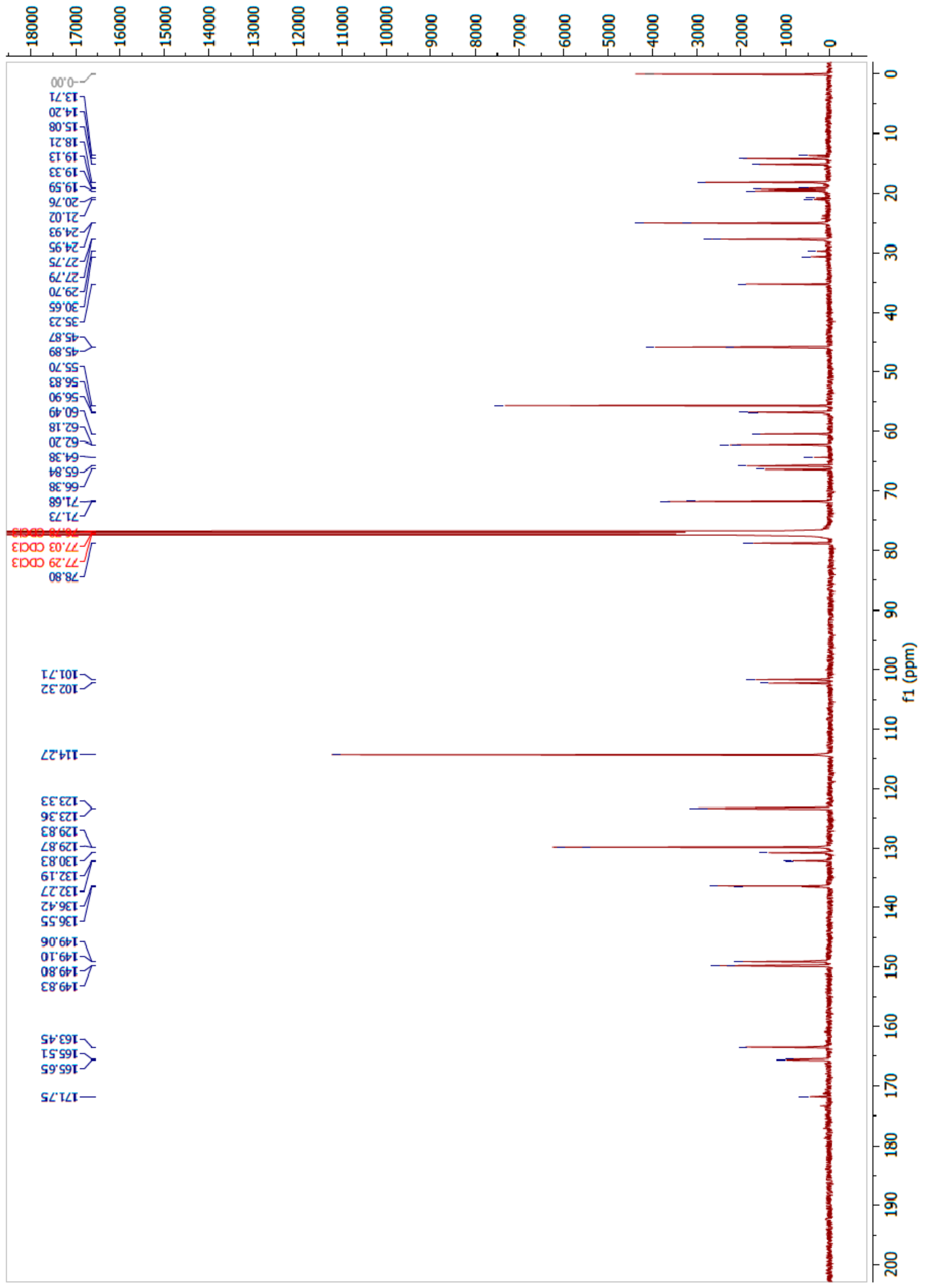

HRMS of 4-methoxy-N-((2R)-1-oxo-1-(((tetrahydro-2H-pyran-2-yl)oxy)-l2-azaneyl)pent-4-yn-2-yl)-N-(pyridin-3-ylmethyl)benzenesulfonamide **(16).**

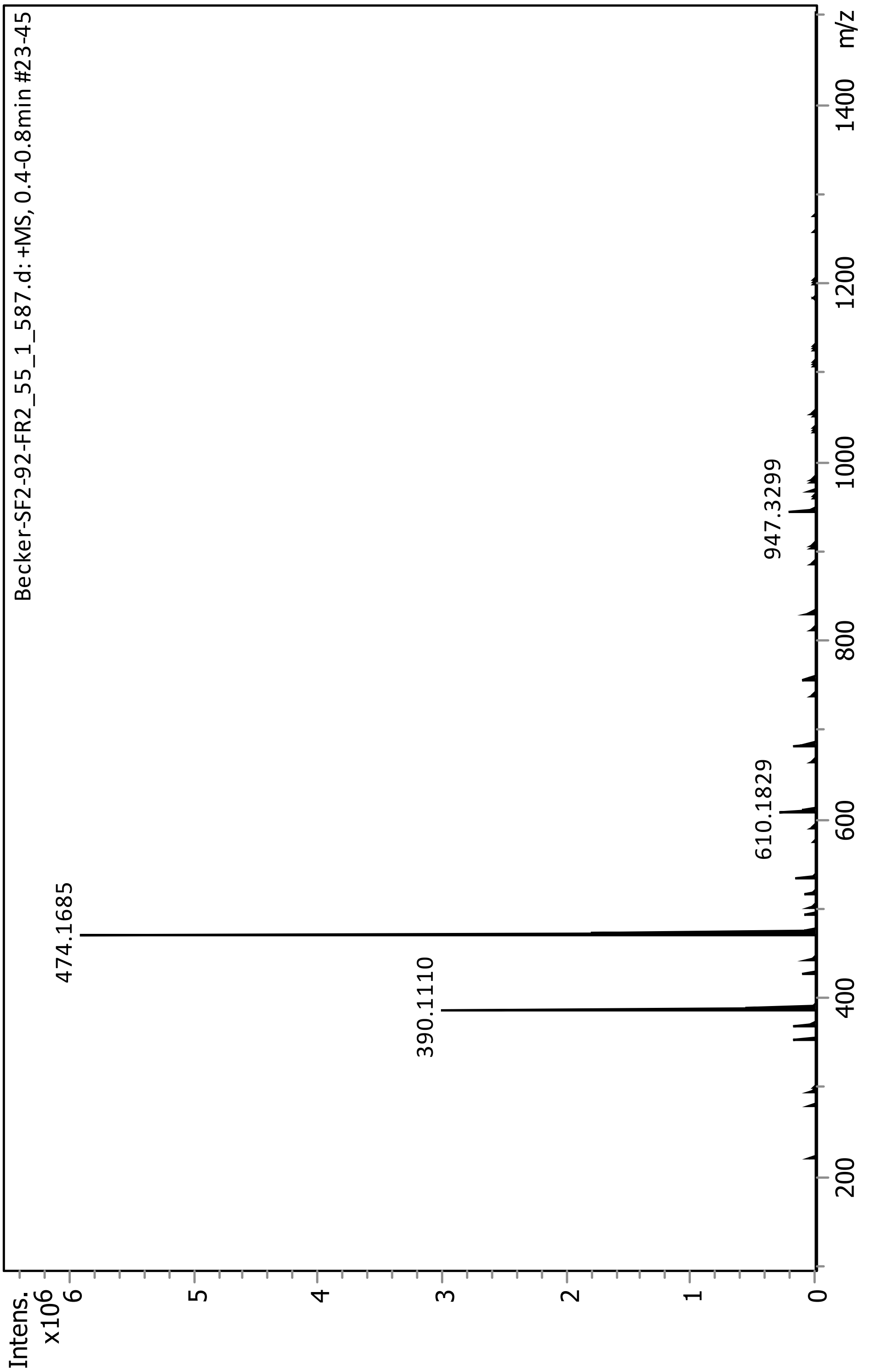

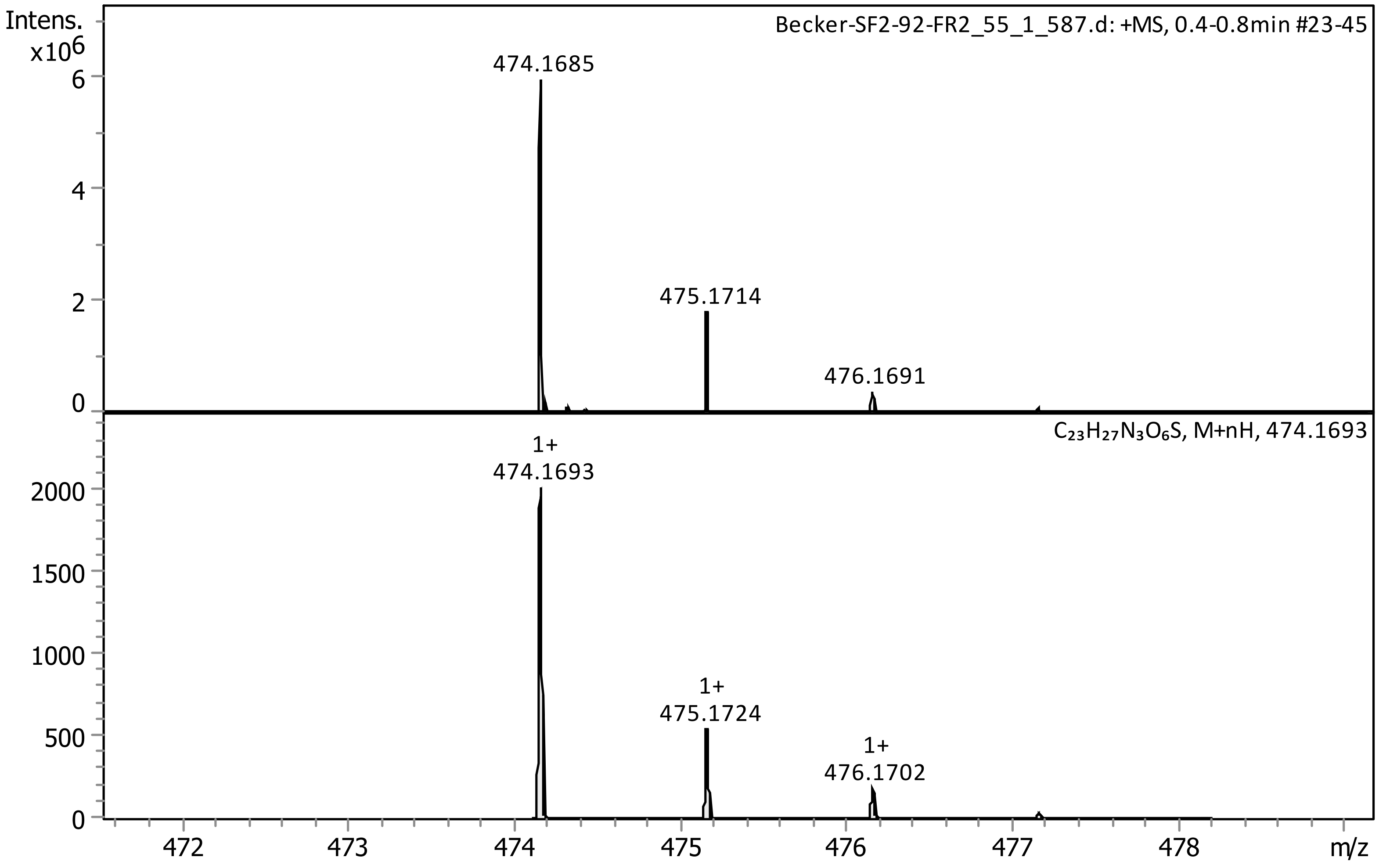
Intens.
x10^6
6
4
2
0
Becker-SF2-92-FR2_55_1_587.d: +MS, 0.4-0.8min #23-45
474.1685
475.1714
476.1691
C₂₃H₂₇N₃O₆S, M+nH, 474.1693
1+
474.1693
1+
475.1724
1+
476.1702
2000
1500
1000
500
0
472
473
474
475
476
477
478
m/z

HPLC of THP-protected 1,4-Click Isomer **18.**

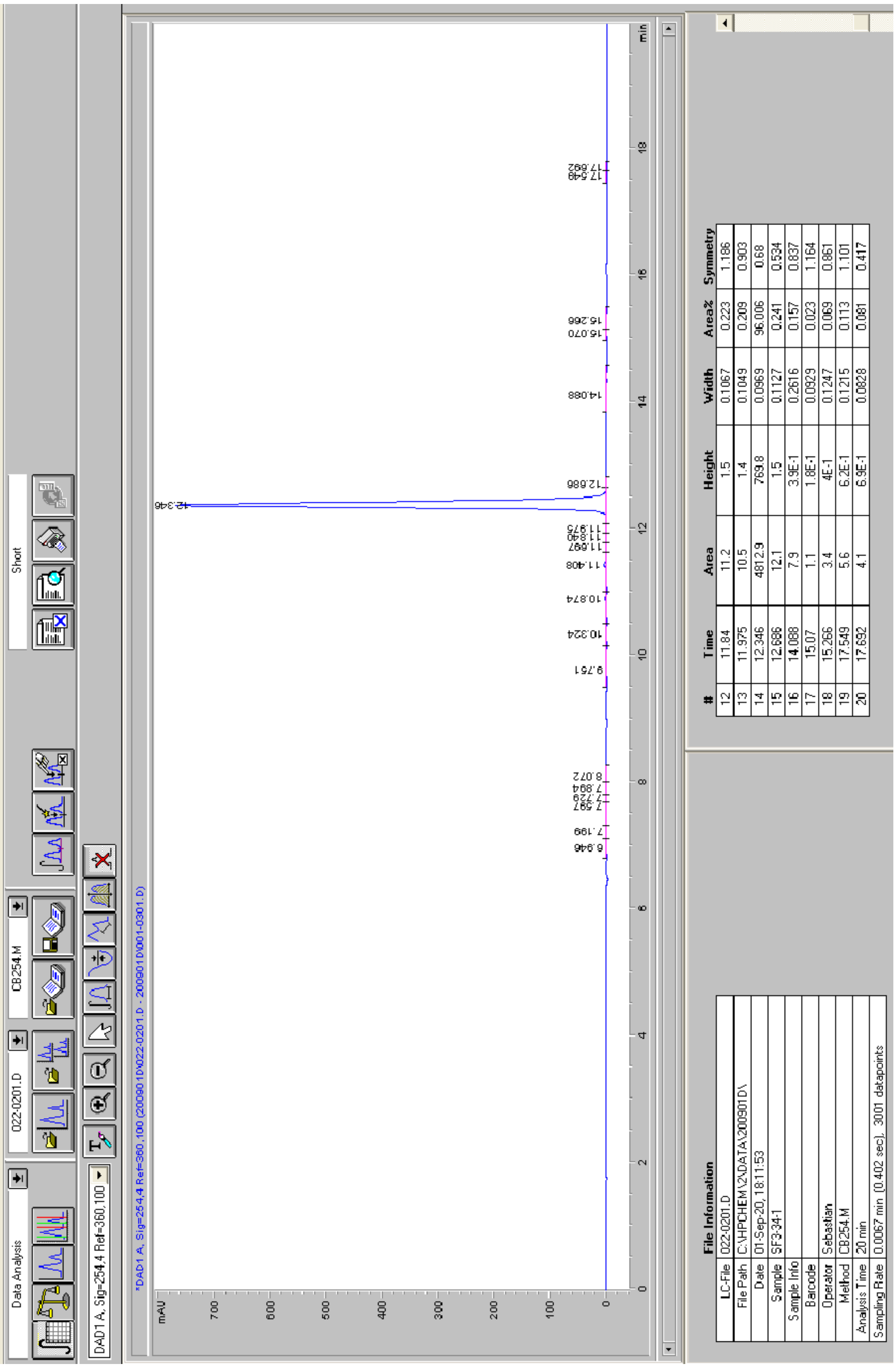

#	Time	Area	Height	Width	Area%	Symmetry
12	11.84	11.2	1.5	0.1067	0.223	1.186
13	11.975	10.5	1.4	0.1049	0.209	0.903
14	12.346	4812.9	769.8	0.0969	96.006	0.68
15	12.686	12.1	1.5	0.1127	0.241	0.534
16	14.088	7.9	3.9E-1	0.2616	0.157	0.837
17	15.07	1.1	1.8E-1	0.0929	0.023	1.164
18	15.266	3.4	4E-1	0.1247	0.069	0.861
19	17.549	5.6	6.2E-1	0.1215	0.113	1.101
20	17.692	4.1	6.9E-1	0.0828	0.081	0.417

File Information	
LC-File	022-0201.D
File Path	C:\HPCHEM\2\DATA\200901D\
Date	01-Sep-20, 18:11:53
Sample Info	SF3-34-1
Barcode	
Operator	Sebastian
Method	CB254.M
Analysis Time	20 min
Sampling Rate	0.0067 min (0.402 sec), 3001 datapoints

^{1}H NMR of THP-protected 1,4-Click Isomer **18.**

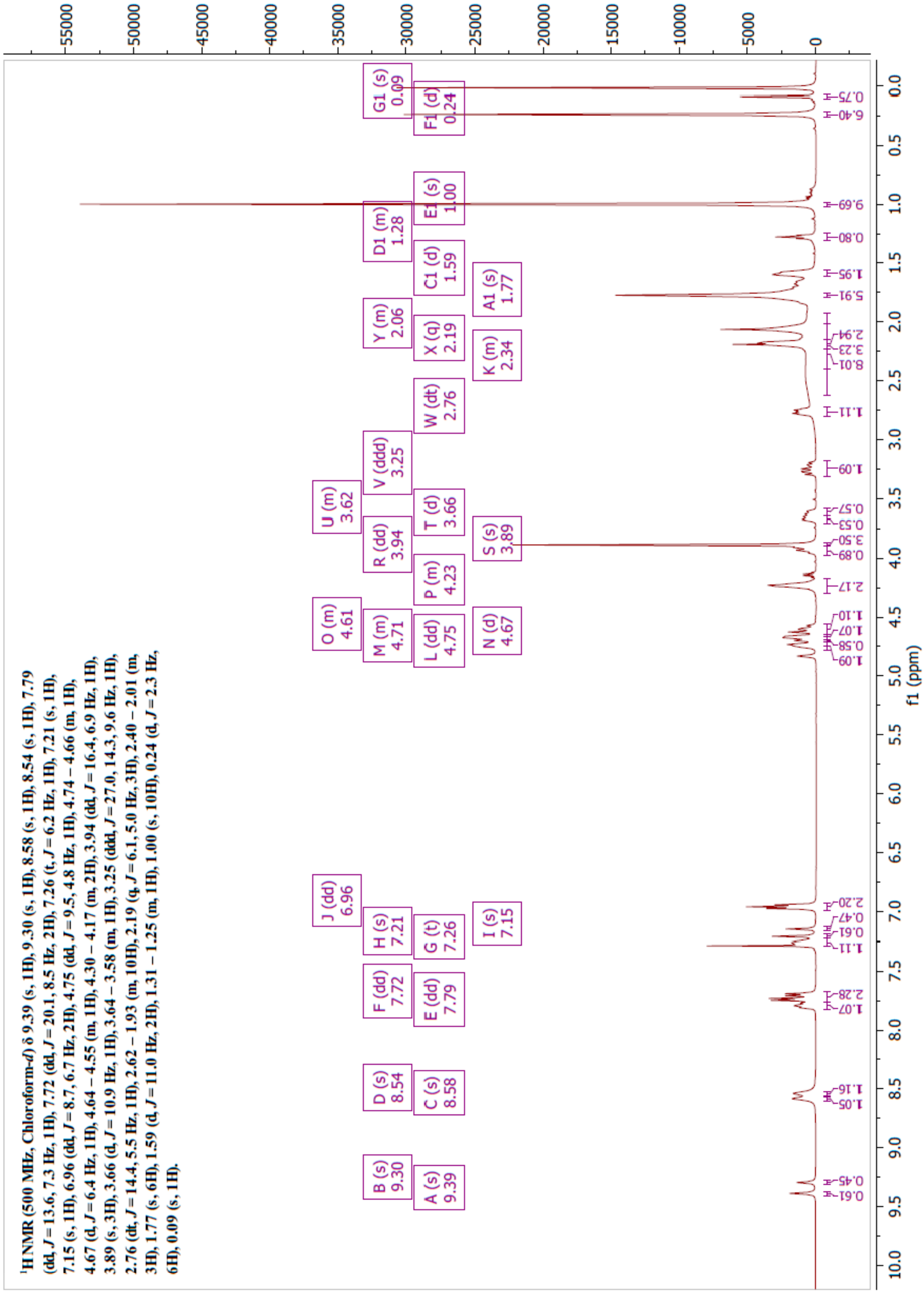

^{13}C NMR of THP-protected 1,4-Click Isomer **18.**

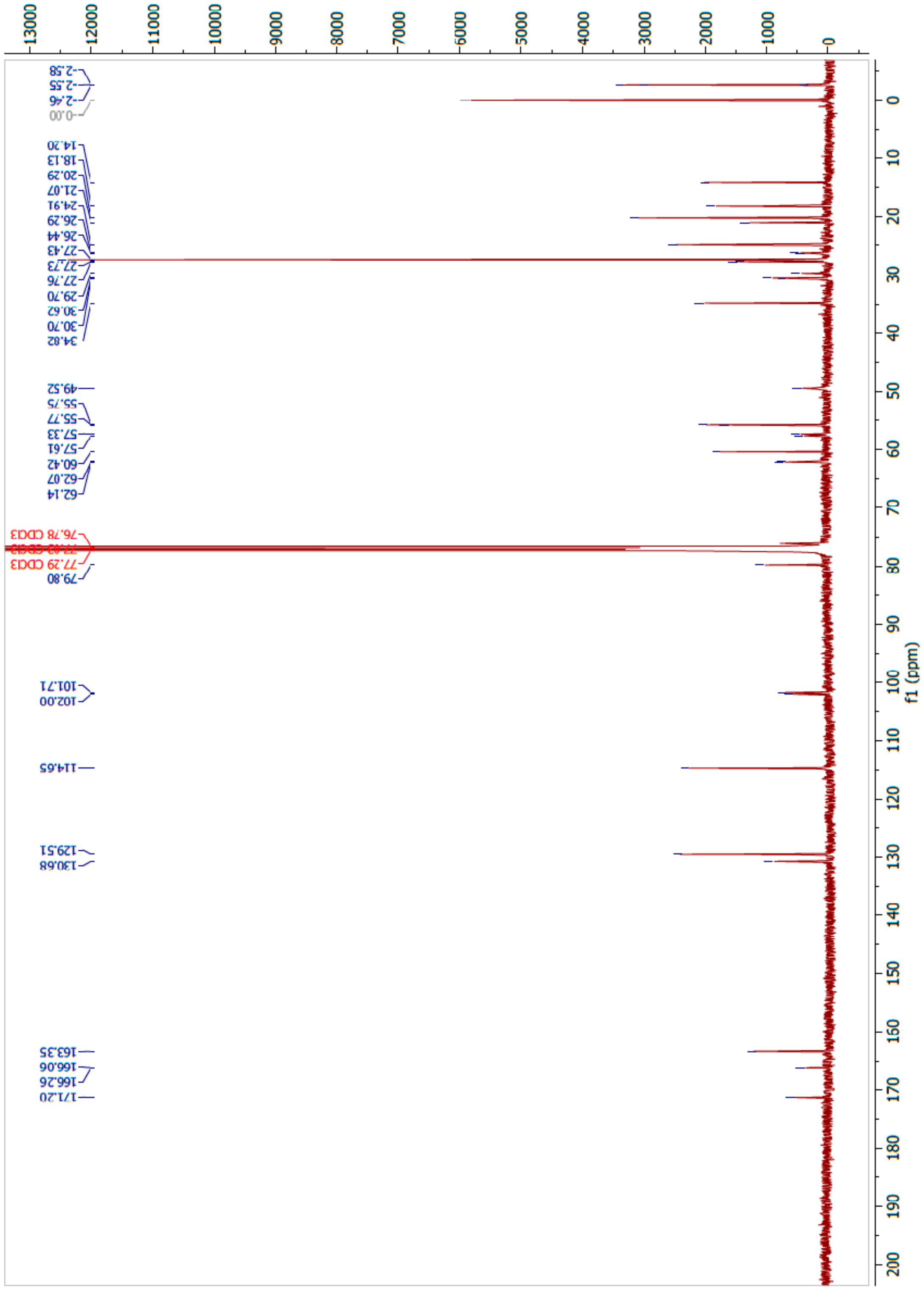

HRMS of THP-protected 1,4-Click Isomer **18.**

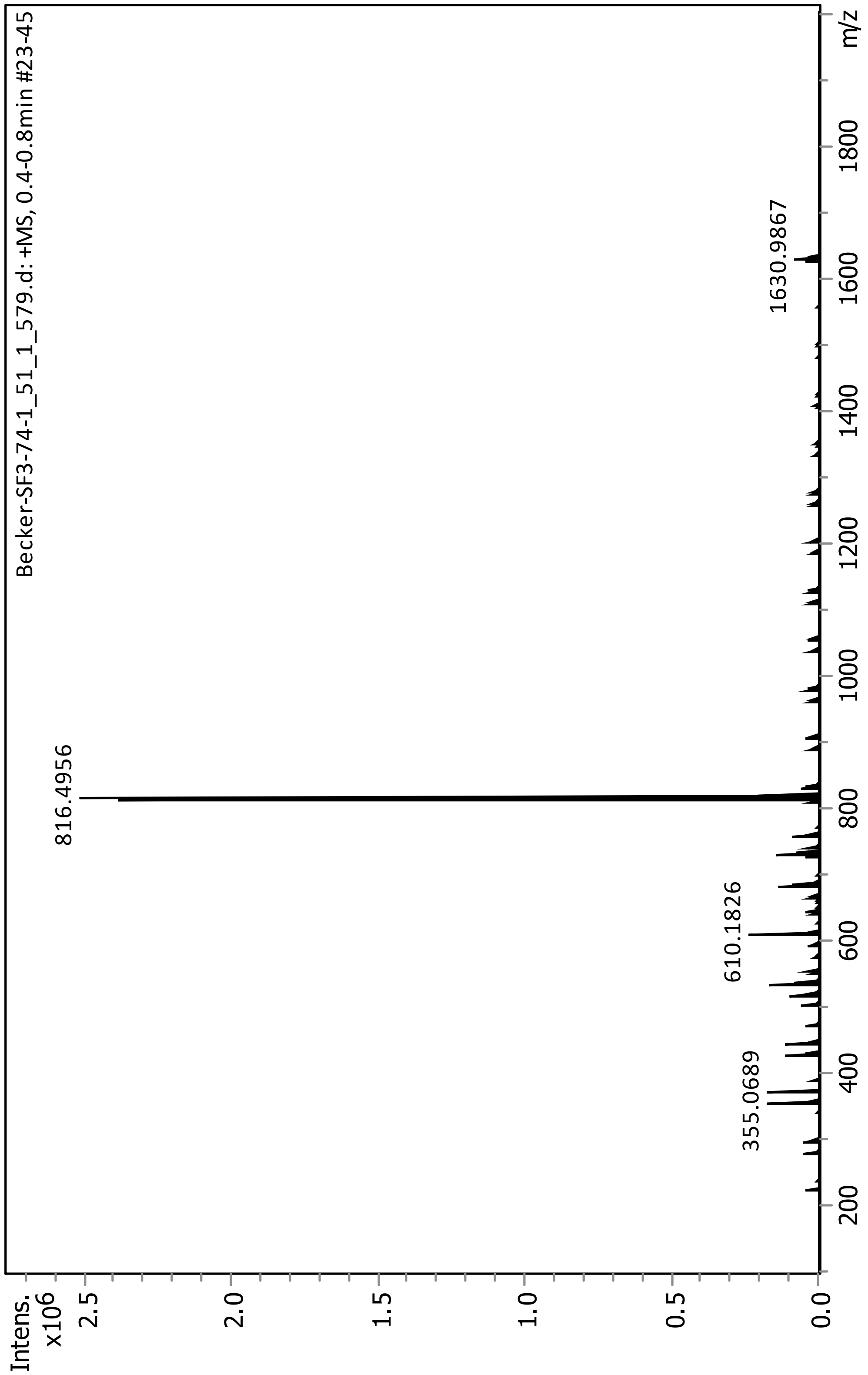

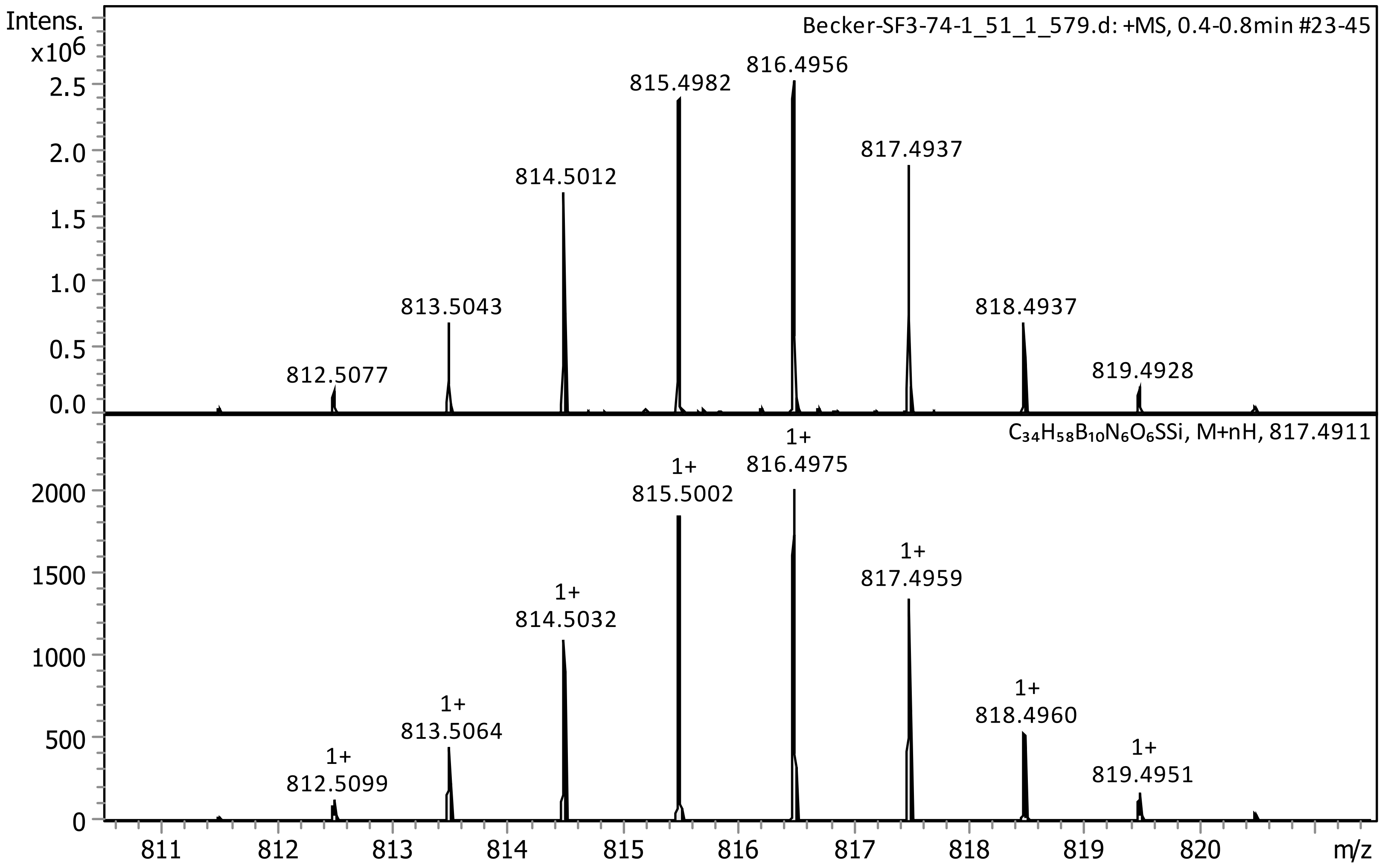
Intens.
x10^6
Becker-SF3-74-1_51_1_579.d: +MS, 0.4-0.8min #23-45
812.5077
813.5043
814.5012
815.4982
816.4956
817.4937
818.4937
819.4928
C34H58B10N6O6SSi, M+nH, 817.4911
1+ 812.5099
1+ 813.5064
1+ 814.5032
1+ 815.5002
1+ 816.4975
1+ 817.4959
1+ 818.4960
1+ 819.4951
m/z

HPLC of Hydroxamic Acid 1,4-triazole **4.**

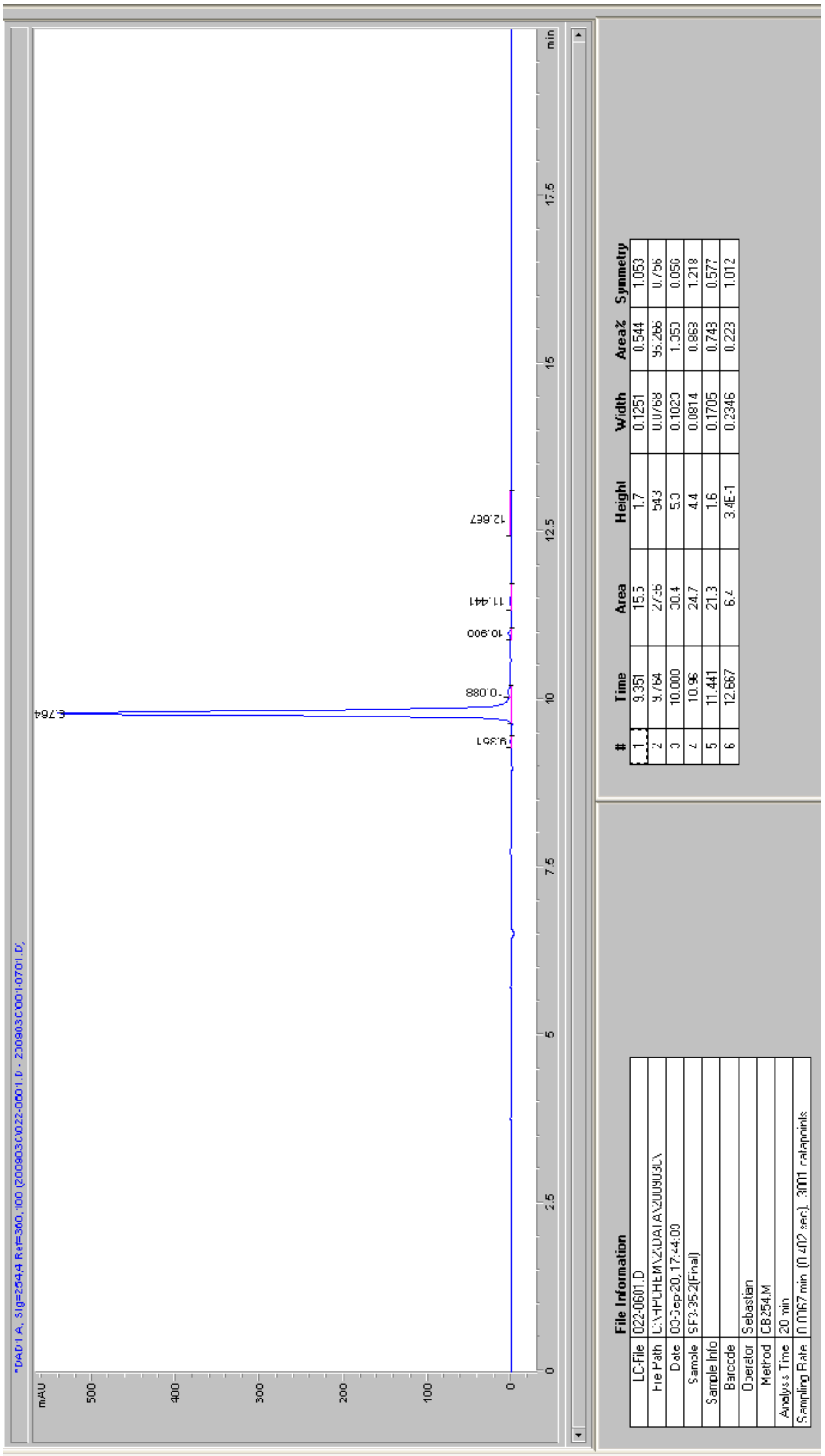

File Information	
LC-File	022-0601.D
File Path	C:\HPCHEM\2\DATA\200903C\
Date	03-Sep-20, 17:44:09
Sample	SF3-35-2(Final)
Sample Info	
Barcode	
Operator	Sebastian
Method	CB254.M
Analysis Time	20 min
Sampling Rate	0.0067 min (0.402 sec), 3001 datapoints

#	Time	Area	Height	Width	Area%	Symmetry
1	9.351	15.5	1.7	0.1251	0.544	1.053
2	9.764	2736	543	0.0768	95.266	0.756
3	10.000	30.4	5.0	0.1020	1.350	0.056
4	10.96	24.7	4.4	0.0814	0.863	1.218
5	11.441	21.3	1.6	0.1705	0.743	0.577
6	12.667	6.4	3.4E-1	0.2346	0.223	1.012

^{1}H NMR of Hydroxamic Acid 1,4-triazole **4.**

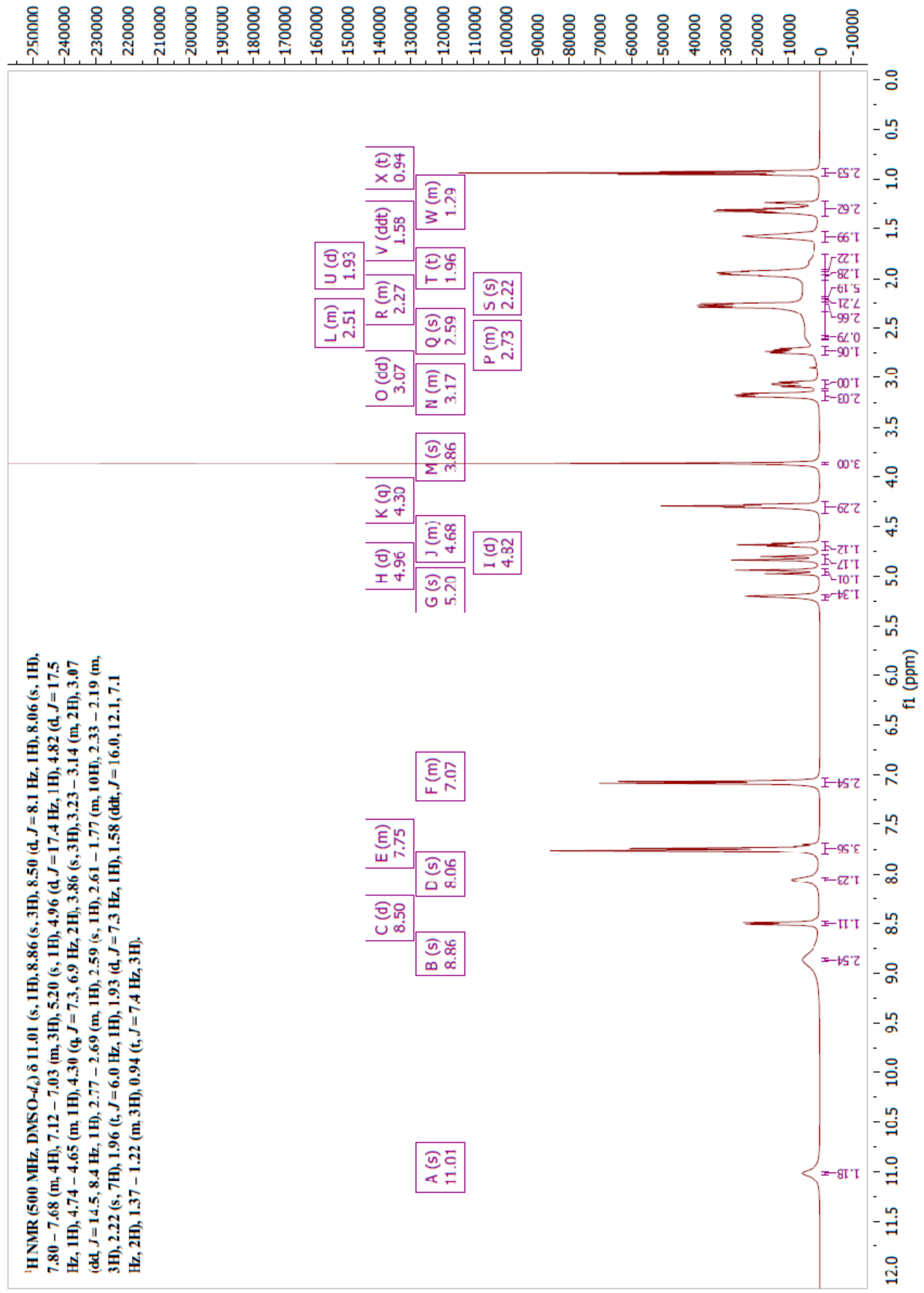

^{13}C NMR of Hydroxamic Acid 1,4-triazole **4.**

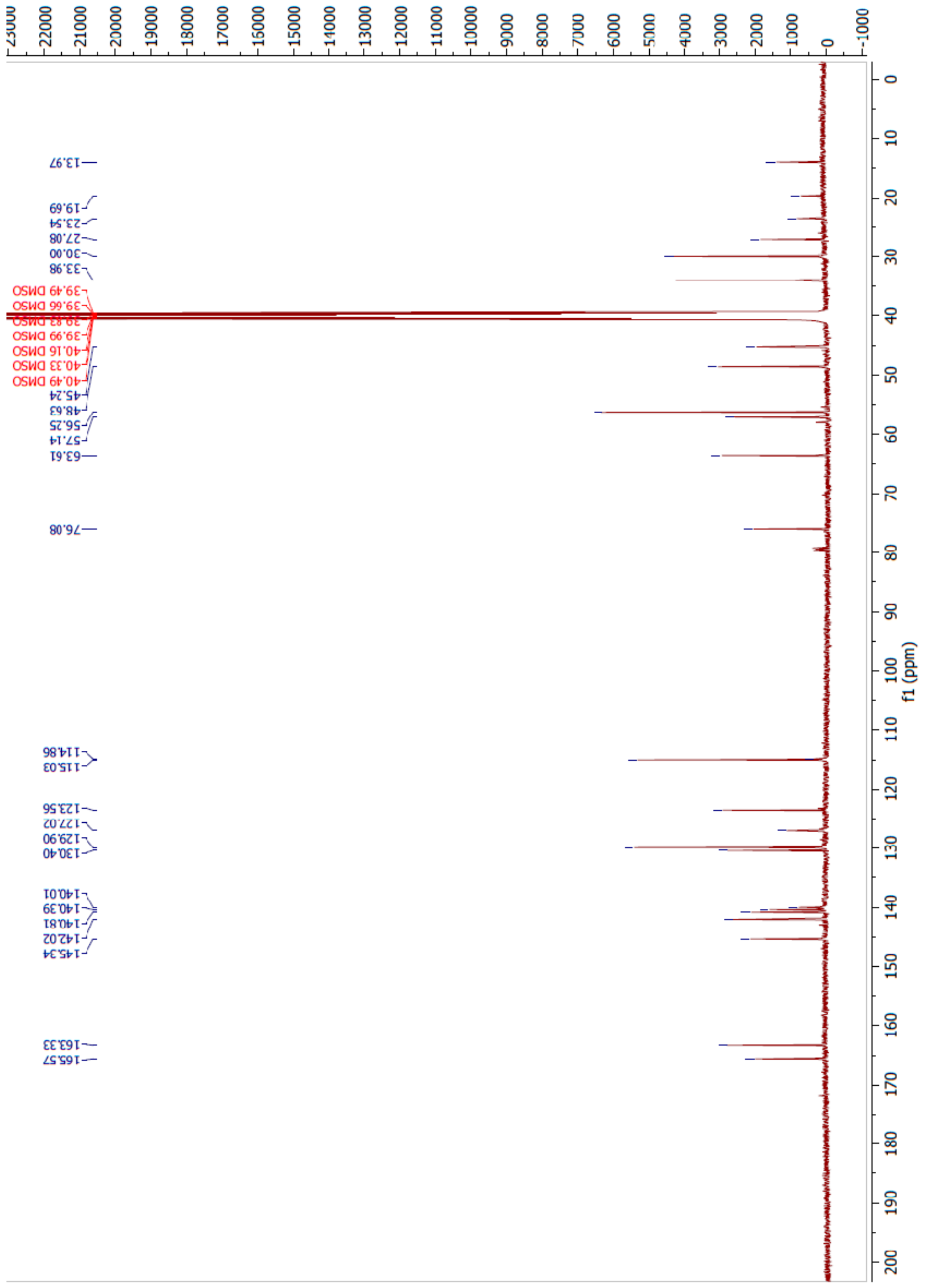

HRMS of Hydroxamic Acid 1,4-triazole **4.**

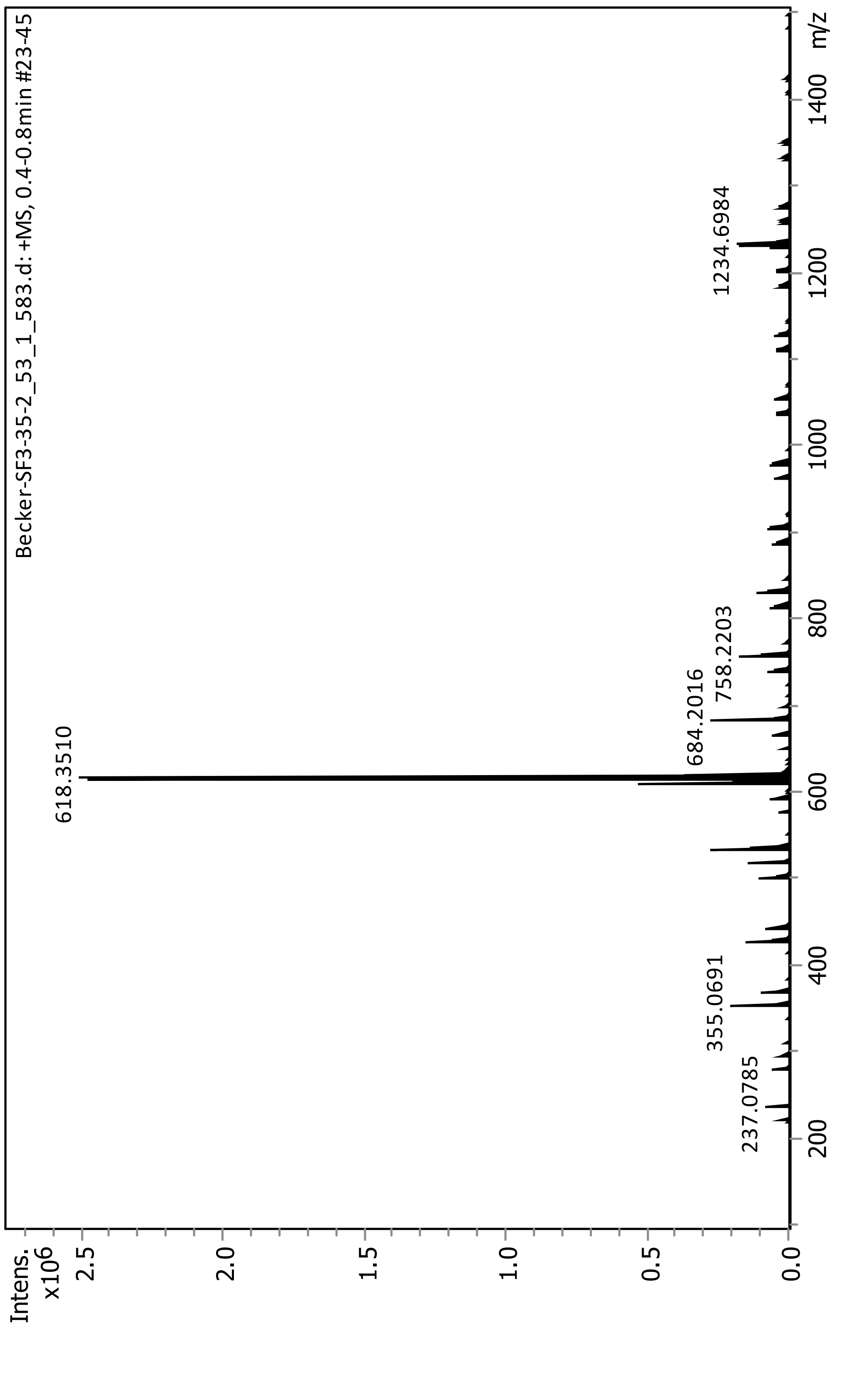

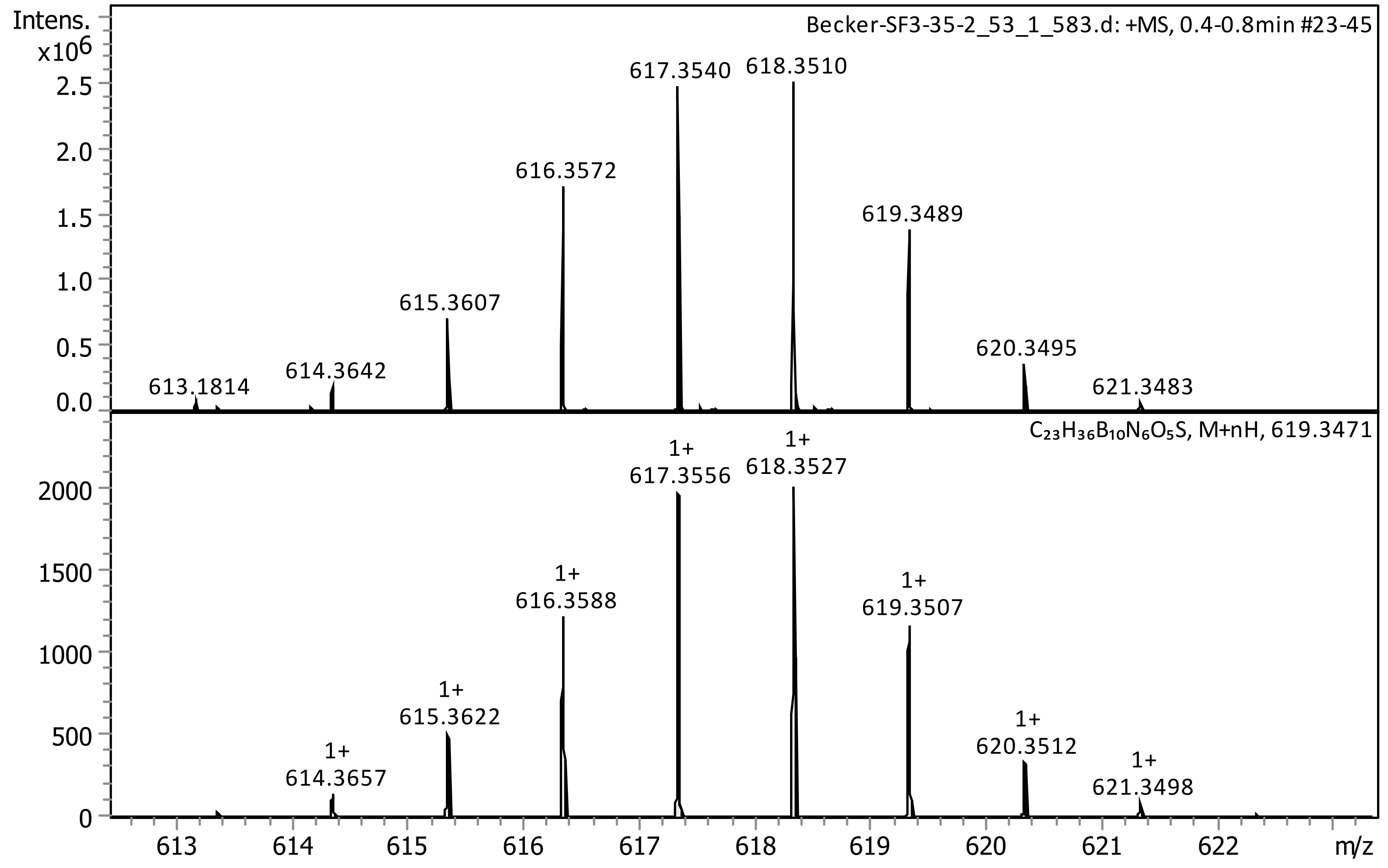

Intens.
x10^6
Becker-SF3-35-2_53_1_583.d: +MS, 0.4-0.8min #23-45
613.1814
614.3642
615.3607
616.3572
617.3540
618.3510
619.3489
620.3495
621.3483
C23H36B10N6O5S, M+nH, 619.3471
1+ 614.3657
1+ 615.3622
1+ 616.3588
1+ 617.3556
1+ 618.3527
1+ 619.3507
1+ 620.3512
1+ 621.3498
m/z

Figure S1a. Superposition of BNCT Agent **20** and SC-74020 compound **19** in 1HOV (MMP-2).

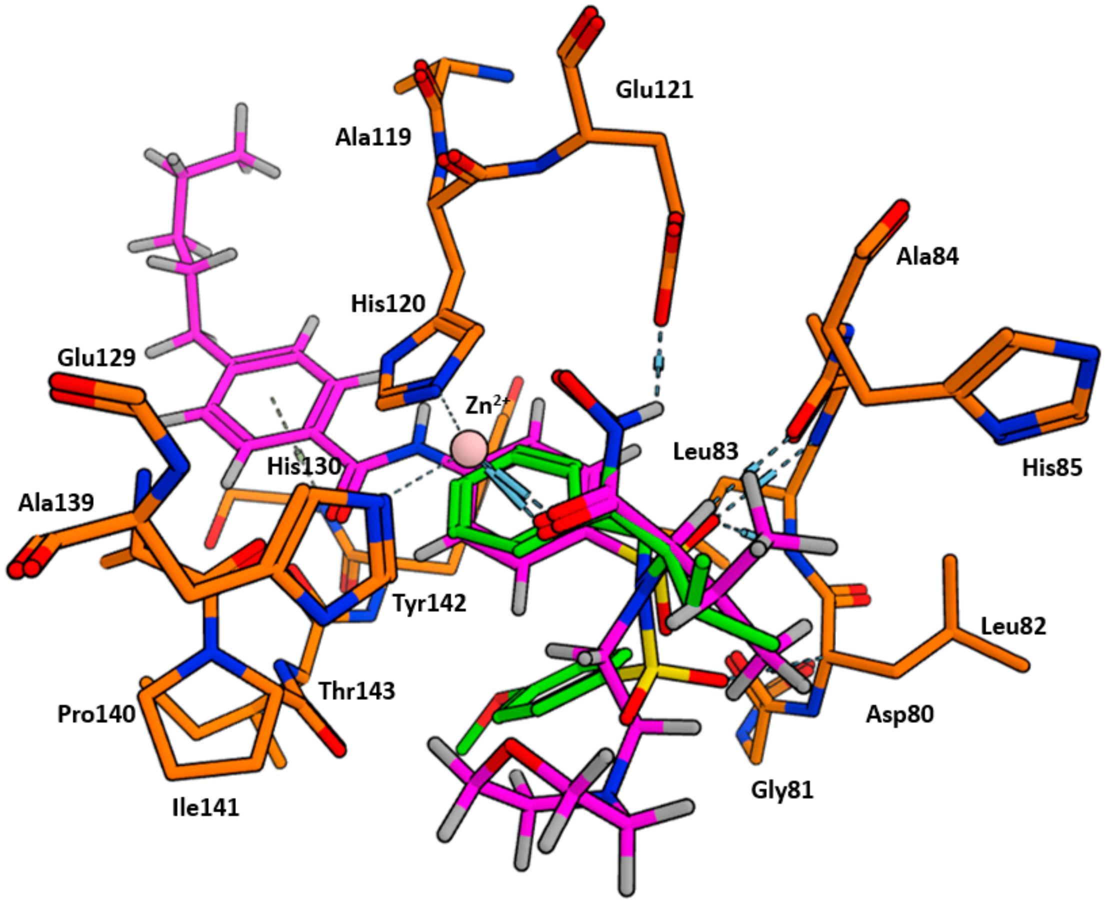

Figure S1b. Ligand interactions of 1HOV (MMP-2) with the bound ligand, BNCT Agent **20**, and Zn^{2+}.

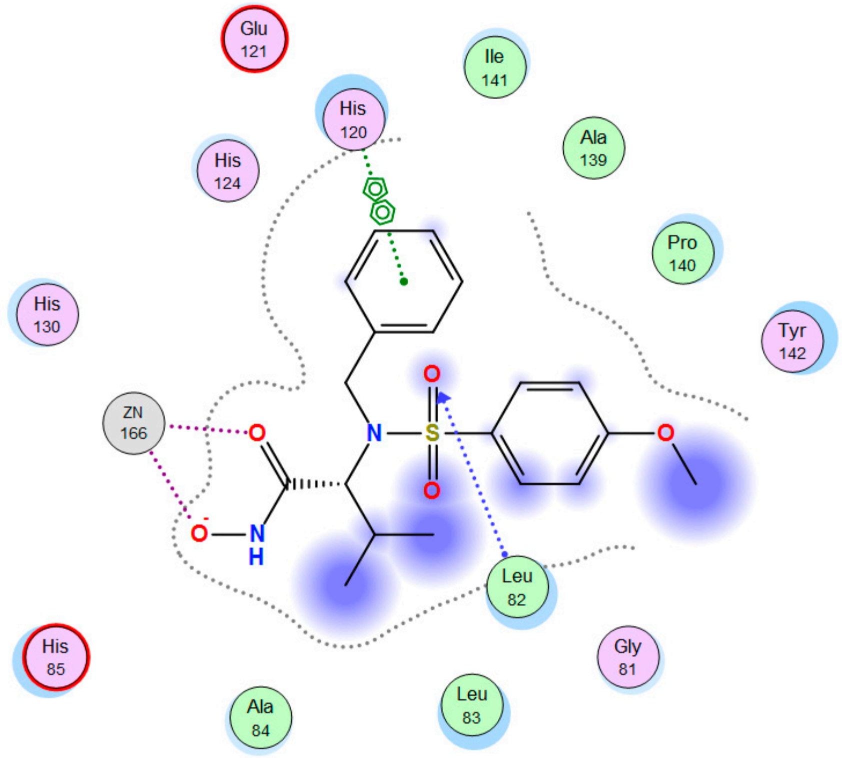

Figure S2a. Superposition of 1,4-click isomer **21** and SC-74020 compound **19** in 1HOV (MMP-2).

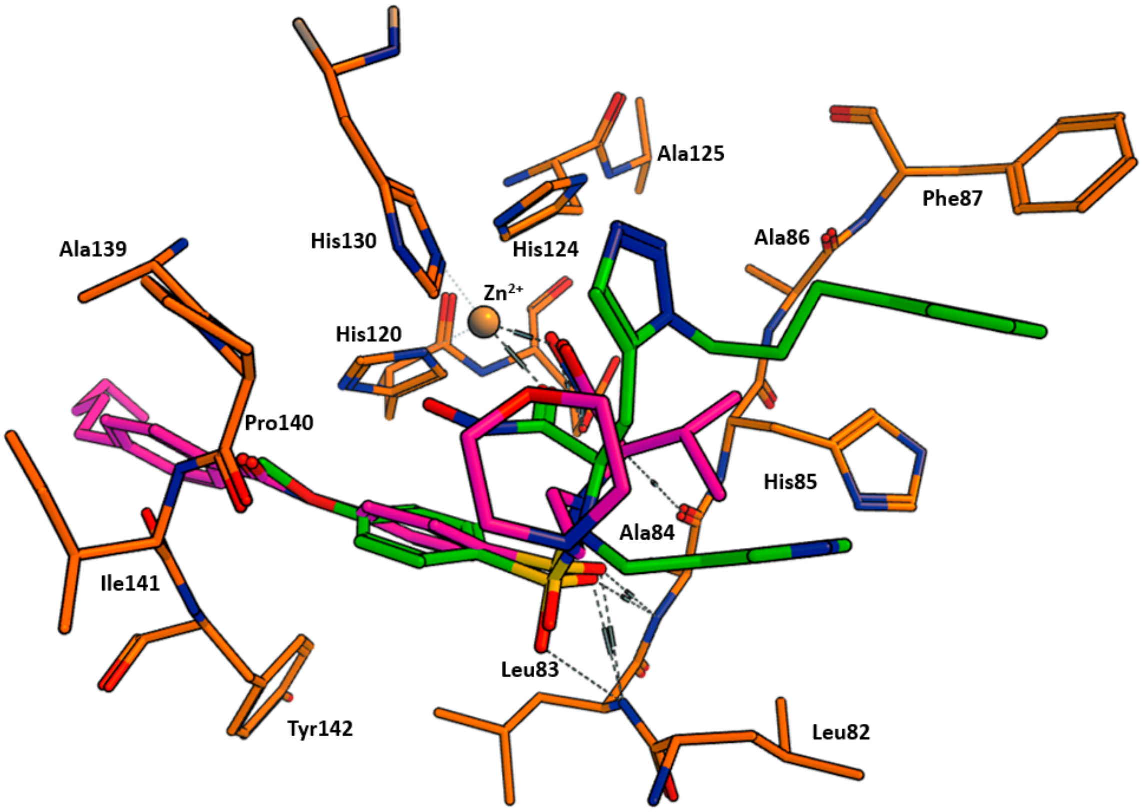

Figure S2b. Ligand interactions of 1HOV (MMP-2) with the bound ligand, 1,4-click isomer **21**, and Zn^{2+}.

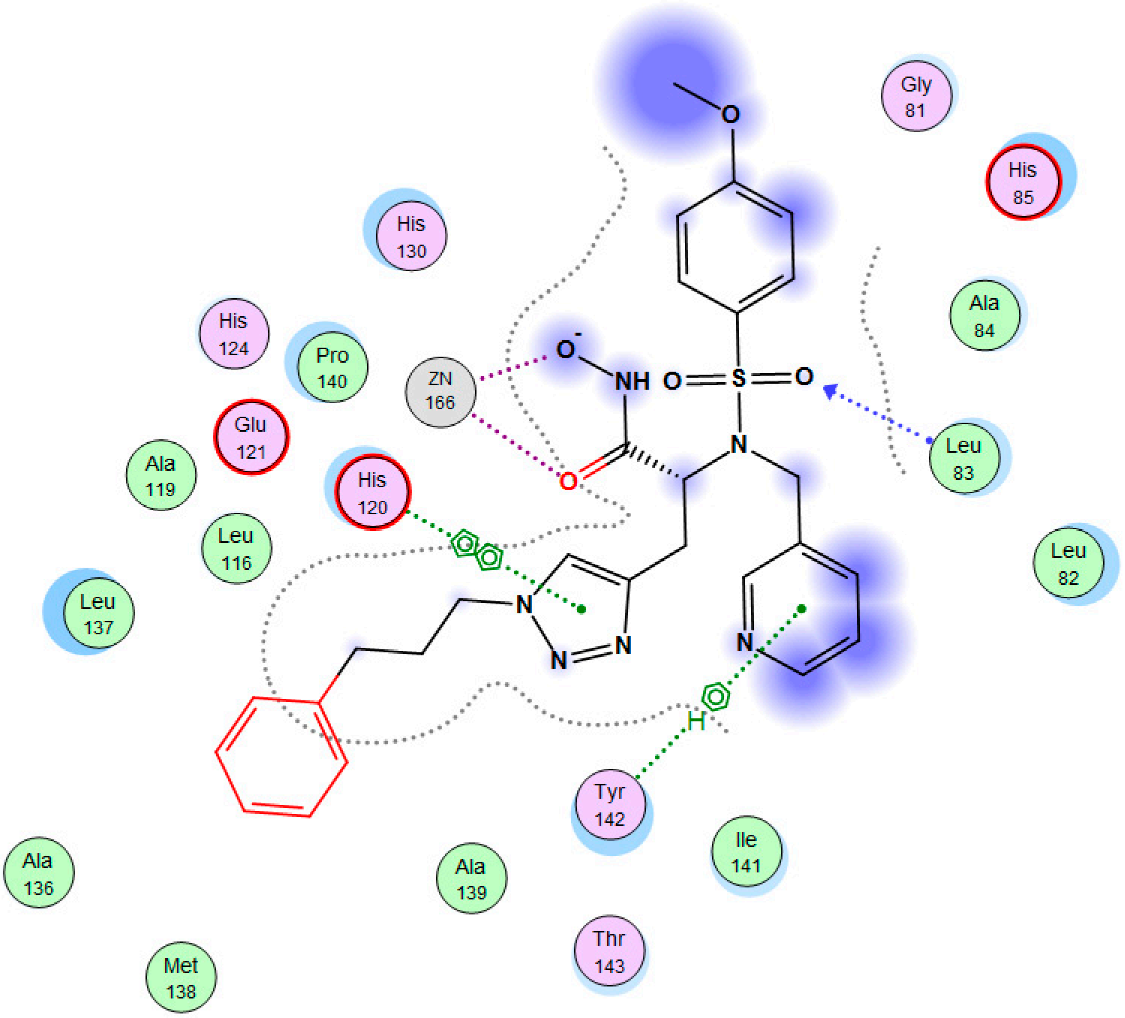

PDB coordinates for docked compounds are available

- **PDB of BNCT Agent 20 Docked in 1HOV.pdb**
- **PDB of BNCT Agent 20 and SC-74020 compound 19 Docked in 1HOV.pdb**
- **PDB of 1,4-Click Isomer 21 Docked in 1HOV.pdb**
- **PDB of 1,4-Click Isomer 21 and SC-74020 compound 19 Docked in 1HOV.pdb**

Compound **3** (SF-4-6A), carborane conjugated MMP inhibitor

Our standardized experimental methods are online at the following URL,

https://1458ab30-7501-42df-8c2e-ff59d20cecb7.filesusr.com/ugd/ddd07a_cbe194d92fd14397a5db1690d68a185c.pdf , which appears on the subsequent pages of this Supplemental Information packet.

The minimum requirement for our screening protocol of Boron compound samples (B-coms) for BNCT is water (and/or DMSO)-soluble 30 mg for first look screening of experiments 1, 2 and 3. Whole serial steps are as follows. Compound **3** (SF-4-6A), was eliminated from further screening at step 2 due to its toxicity.

1. Solubility in a physiological condition
2. Cell toxicity; IC_{50}
3. Cellular BNCT (in-vitro BNCT)
4. Bio-distribution study (neutron induced boron autoradiography)
5. Animal BNCT (in-vivo BNCT)
6. Pre-clinical study

Experimental summary for carborane-containing MMP ligand **3** (SF-4-6A)

Compound **3** (SF-4-6A, abbreviated SF46A) information:

1) The carborane cluster has been prepared with enriched ^{10}B decaborane (>99% ^{10}B)

2) See compound **3** (SF-4-6A) in manuscript and experimental.

3) Molecular Formula: $C_{15}H_{30}{}^{10}B_{10}N_2O_5S$ boron content=10x10/450.32=22.2%

4) Molecular Weight: 450.32 g/mol (calculated based on enriched-^{10}B)

	Experiment 1	Experiment 2	Experiment 3
sample	**3** (SF46A)	**3** (SF46A)	**3** (SF46A)
Solvent (mg)	20mg	20mg	20mg
Dissolved in DMSO	1ml	1ml	1ml
Add (x μl) into culture dish	50μl	100	200<br>=4mg/ml SF46A<br>=0.8ppm^{10}B/ml
Solubility	Completely and easily dissolved	Completely and easily dissolved	Completely and easily dissolved

Filtering for sterilization through 0.022 m pore	Easily done	Easily done	Easily done
Pulse 23:20	50µ	100µ	200µ
Turbidity after adding	+ (clouded)	++ (strongly clouded)	+++ (strongly clouded)
Cell condition 3hrs after pulsation 01:50	Round shaped (toxic)	Detached from the bottom of the culture dish (toxic)	Detached from the bottom of the culture dish (toxic)
04:20 incubation stop and cell condition	Floating from the bottom of the culture dish (toxic)	Floating from the bottom of the culture dish (toxic)	Floating from the bottom of the culture dish (toxic)
ppm Boron in medium	22.2	44.4	66.6

*20mgx0.1x0.1x.22.2%=44.4µg/ml

IC50

Boron compound	n	$0.5x(1/2)^{n-1}$	A mg/ml	$IC_{50}=Ax0.5x(1/2)^{n-1}$ mg/ml
SF-4-6A	10.5	$6.91x10^{-4}$	20	$1.38x10^{-2}$ ($3.07x10^{-5}$ mM) ($3.07x10^{-3}$ ppm ^{10}B)
DSMO	5	$3.13x10^{-2}$		$3.13x10^{-1}$

Water Solubility Determination of 1,4-click triazole **1**

Solvent	1,4-Click Triazole **1**			
	Sample	Concentration (mg/mL)	Trial #1	Trial #2
			Peak Area	Peak Area
Acetonitrile	**A-0**	2.00	9964.6	9772.6
	**A-1**	1.00	5061.8	4915.7
	**A-2**	0.50	2534.7	2473.0
	**A-3**	0.25	1276.0	1250.7
	**A-4**	0.10	503.0	478.3
	**A-5**	0.05	247.9	233.2
	**A-6**	0.01	8.3	8.8
Nanopure H_2O	**A-Aq**	Unknown	44	23.3

Figure S3A. Raw data from HPLC analysis to determine drug water solubility of 1,4-click triazole **1**.

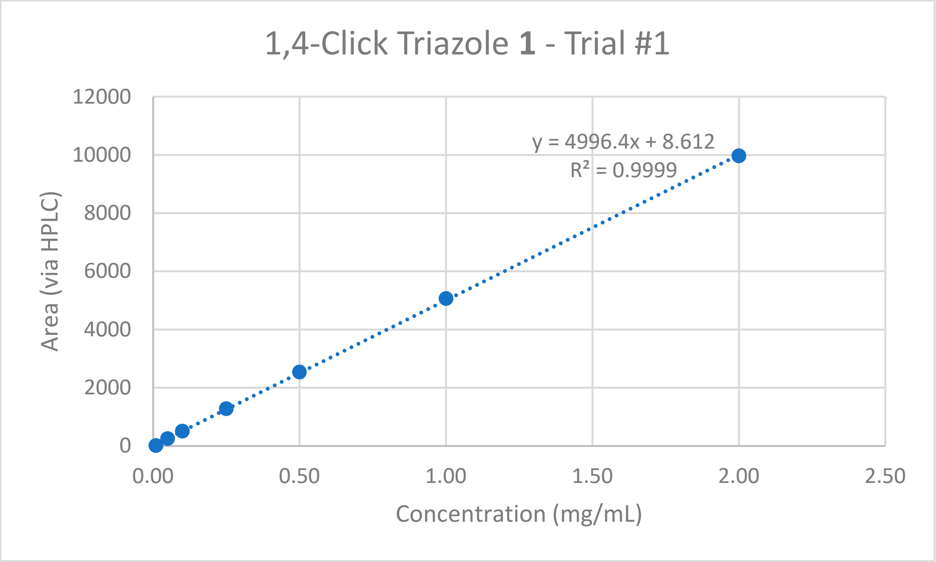

Figure S3B. Calibration graph (Trial 1) generated via HPLC analysis to determine drug water solubility of 1,4-click triazole **1**.

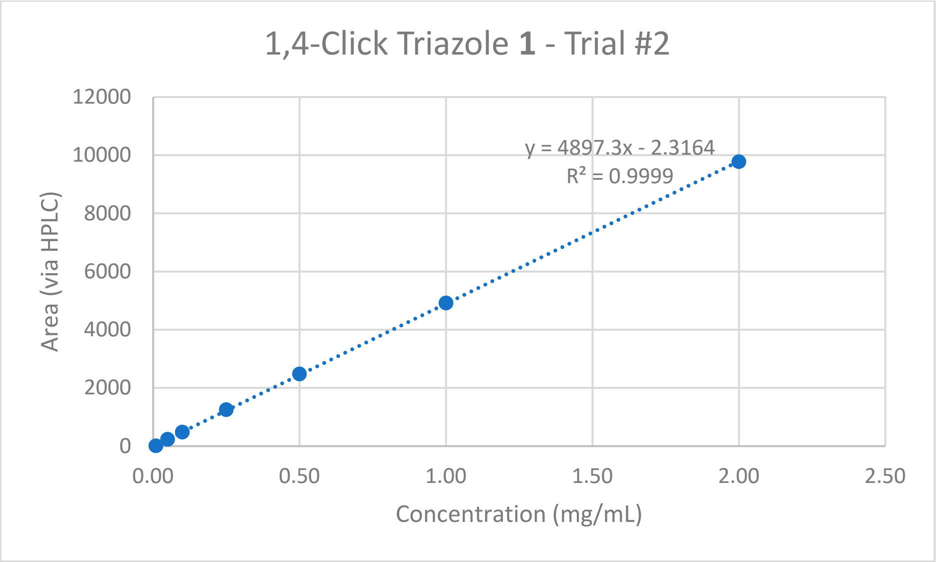

Figure S3C. Calibration graph (Trial 2) generated via HPLC analysis to determine drug water solubility of 1,4-click triazole **1**.

Water Solubility Determination of 1,5-click triazole **2**

Solvent	1,5-Click Triazole **2**			
	Sample	Concentration (mg/mL)	Trial #1	Trial #2
			Peak Area	Peak Area
Acetonitrile	**B-0**	2.00	11789.5	11884.4
	**B-1**	1.00	5930.6	6000.2
	**B-2**	0.50	2975.9	2993.6
	**B-3**	0.25	1473.9	1521.9
	**B-4**	0.10	583.7	625.6
	**B-5**	0.05	91.7	334.8
	**B-6**	0.01	10.6	22.4
Nanopure H_2O	**B-Aq**	Unknown	29.5	20.5

Figure S4A. Raw data generated via HPLC analysis to determine drug water solubility of 1,5-click triazole **2**.

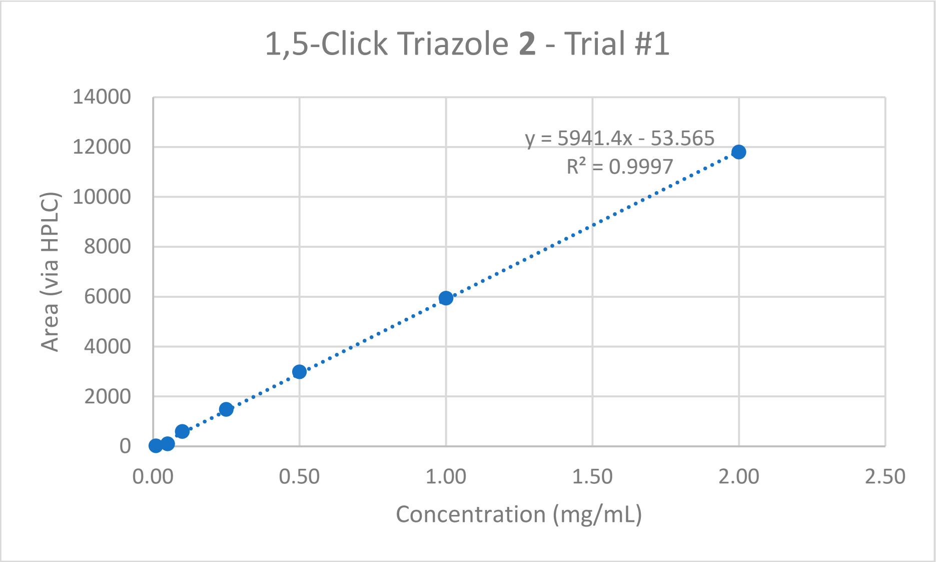

Figure S4B. Calibration graph (Trial 1) generated via HPLC analysis to determine drug water solubility of 1,5-click triazole **2**.

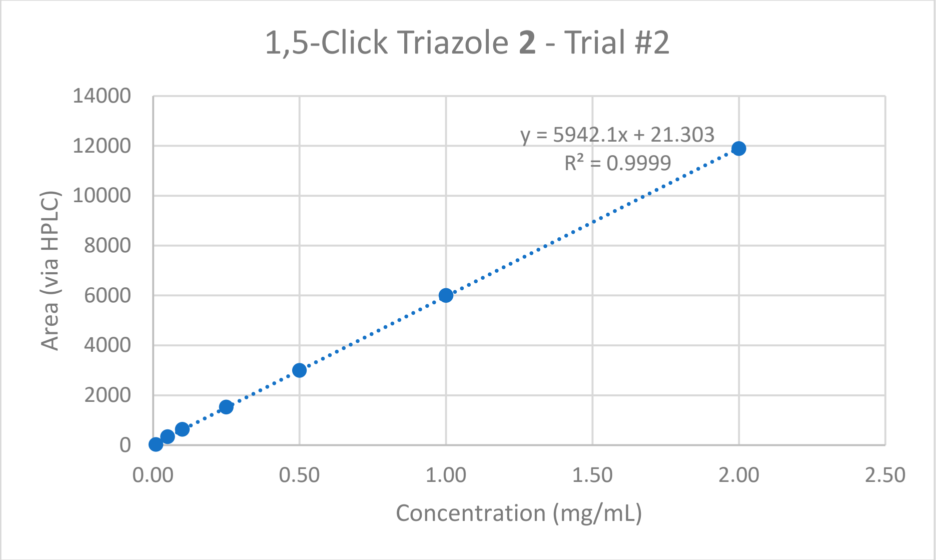

Figure S4C. Calibration graph (Trial 2) generated via HPLC analysis to determine drug water solubility of 1,5-click triazole **2**.

Water Solubility Determination of CGS-27023A analog **3**.

Solvent	CGS-27023A Enriched ^{10}B Analog **3**			
	Sample	Concentration (mg/mL)	Trial #1	Trial #2
			Peak Area	Peak Area
Acetonitrile	**C-0**	2.00	5863.2	5942.2
	**C-1**	1.00	2992.0	3044.7
	**C-2**	0.50	1526.5	1601.9
	**C-3**	0.25	762.2	845.1
	**C-4**	0.10	308.6	351.7
	**C-5**	0.05	154.1	174.0
	**C-6**	0.01	7.0	23.3
Nanopure H_2O	**C-Aq**	Unknown	17.8	91.8

Figure S5A. Raw data generated via HPLC analysis to determine drug water solubility of CGS-27023A analog **3**.

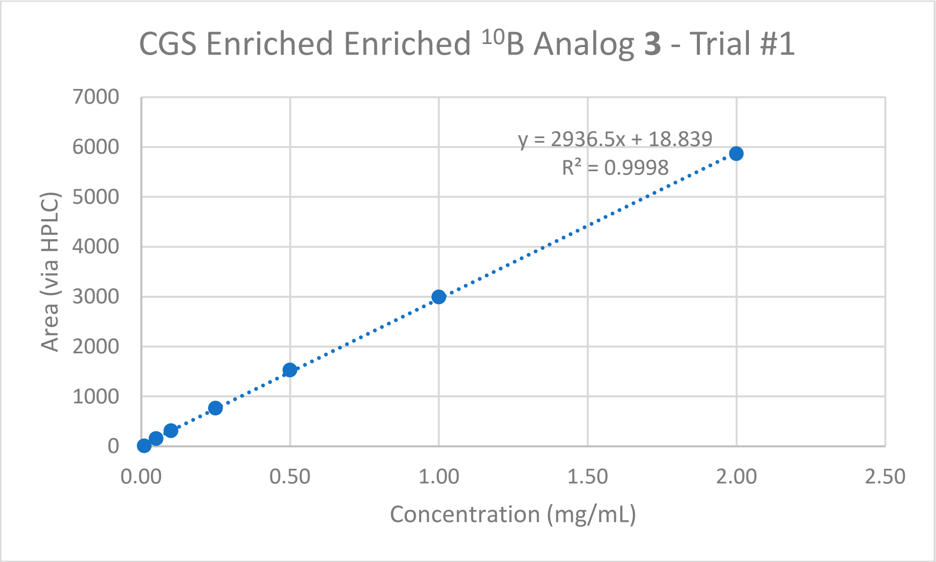

Figure S5B. Calibration graph (Trial 1) generated via HPLC analysis to determine drug water solubility of CGS-27023A analog **3**.

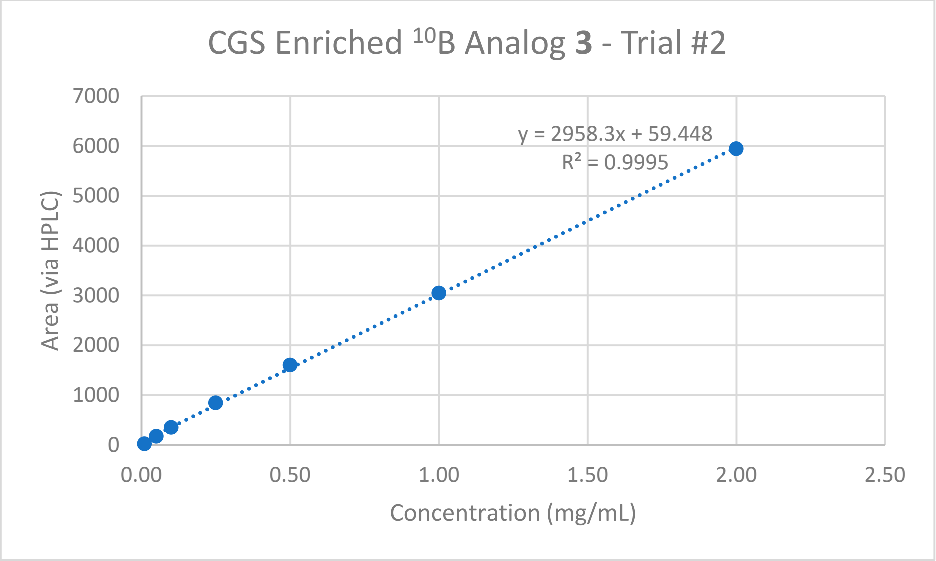

Figure S5C. Calibration graph (Trial 2) generated via HPLC analysis to determine drug water solubility of CGS-27023A analog **3**.

Water Solubility Determination of 1,4-Click triazole **4**.

Solvent	CGS-27023A 1,4-Click Triazole **4**			
	Sample	Concentration (mg/mL)	Trial #1	Trial #2
			Peak Area	Peak Area
Methanol	**D-0**	2.00	6666.0	6821.1
	**D-1**	1.00	3315.6	3381.9
	**D-2**	0.50	1617.4	1635.8
	**D-3**	0.25	799.8	810.5
	**D-4**	0.10	307.3	310.0
	**D-5**	0.05	150.2	156.2
	**D-6**	0.01	5.9	7.5
Nanopure H_2O	**D-Aq**	Unknown	321.1	257.9

Figure S6A. Raw data generated via HPLC analysis to determine drug water solubility of CGS-27023A 1,4-Click triazole **4**.

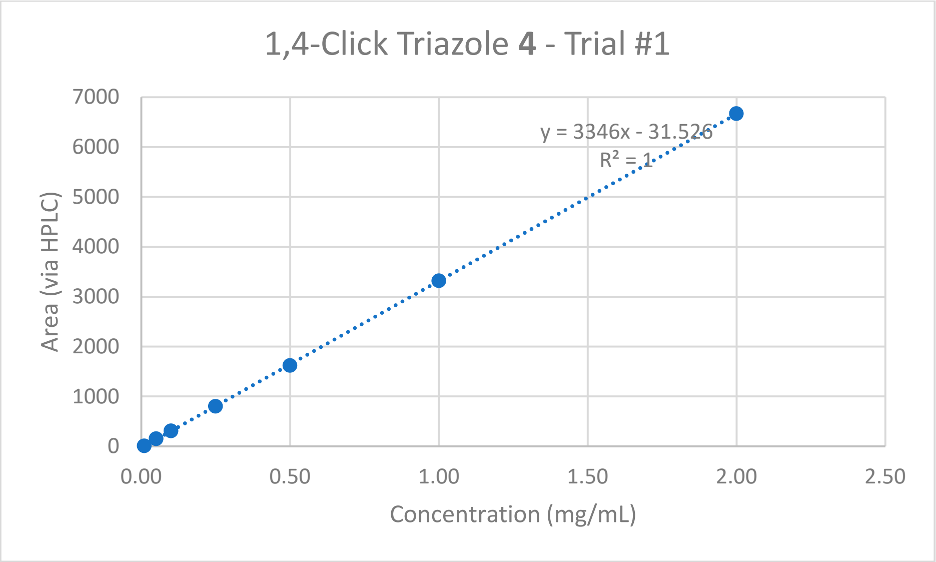

Figure S6B. Calibration graph (Trial 1) generated via HPLC analysis to determine drug water solubility of CGS-27023A 1,4-Click triazole **4**.

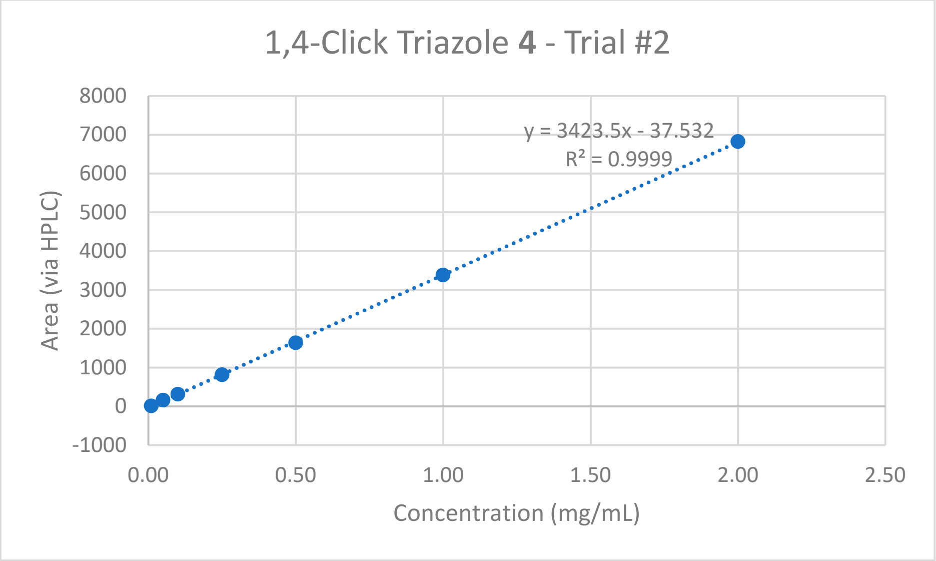

Figure S6C. Calibration graph (Trial 2) generated via HPLC analysis to determine drug water solubility of CGS-27023A 1,4-Click triazole **4**.

U87 MG *delta EGFR* (colony efficiency 4 %)

U87	X (Thermal Fluence)	Y (Survival fraction)	std in SF
control	0.00E+00	1	0.090138782
	9.24E+11	0.758333333	0.038188131
	1.91E+12	0.5	0.066143783
	2.50E+12	0.333333333	0.057735027
BPA(^{10}B 20ppm)	0.00E+00	1	0.070168517
	9.24E+11	0.494382022	0.07784498
	1.91E+12	0.202247191	0.033707865
	2.50E+12	0.179775281	0.03892249
#4 (^{10}B 0.48ppm)	0.00E+00	1	0.021650635
	9.24E+11	0.6125	0.229128785
	1.91E+12	0.2125	0.057282196
	2.50E+12	0.0625	0.021650635

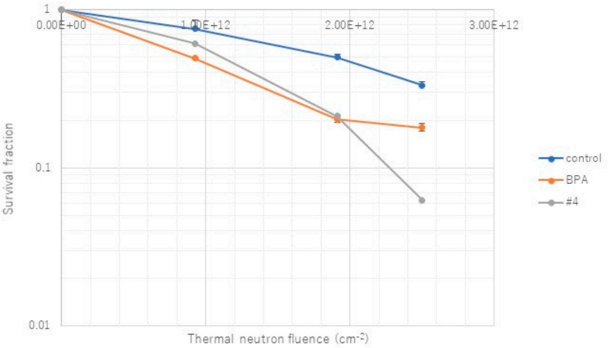

Survival fraction in U87 MG cells. BPA: 20 ppm ^{10}B; compound **4**: 0.48 ppm ^{10}B treated with U87 *delta EGFR* cells for 4 hours

Figure S7a. Survival fraction in U87 MG cells. BPA: 20 ppm ^{10}B; compound **4**: 0.48 ppm ^{10}B treated with U87 delta EGFR cells for 4 hours with raw data and standard deviation values.

SCC 7 (colony efficiency 34 %)

SCC7	X (Thermal Fluence)	Y (Survival fraction)	std in SF
control	0.00E+00	1	0.120983443
	9.24E+11	0.79296875	0.097578094
	1.91E+12	0.62109375	0.025540423
	2.50E+12	0.58984375	0.018835255
BPA (^{10}B 20ppm)	0.00E+00	1	0.195263916
	9.24E+11	0.20824295	0.006507592
	1.91E+12	0.093275488	0.00751432
	2.50E+12	0.054229935	0.009940511
#4(^{10}B 0.48ppm)	0.00E+00	1	0.111704296
	9.24E+11	0.621880998	0.077468085
	1.91E+12	0.548944338	0.008795731
	2.50E+12	0.378119002	0.01198656

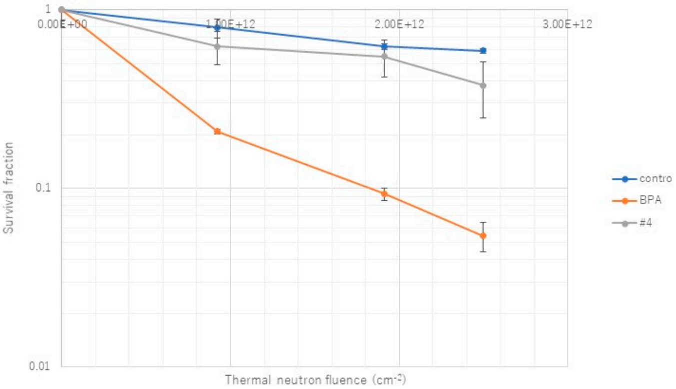

Survival fraction in SCC7 cells. BPA: 20 ppm ^{10}B; compound **4**: 0.48 ppm ^{10}B treated with SCC7 cells for 4 hours.

Figure S7b. Survival fraction in SCC7 cells. BPA: 20 ppm ^{10}B; compound **4**: 0.48 ppm ^{10}B treated with SCC7 cells for 4 hours with raw data and standard deviation values.

<u>Boron concentration measurement by ICP-OES</u>

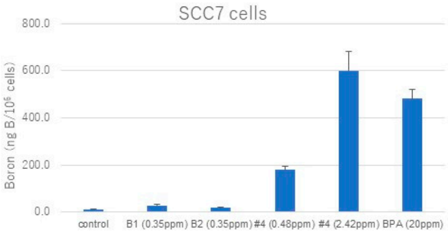

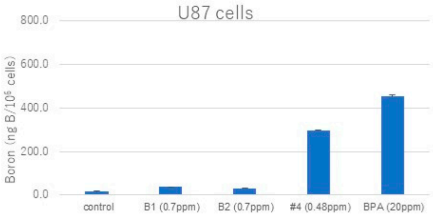

	ng B/106 cell	
Sample ID	Av.	SD
control	12.6	1.1
B1 (0.35ppm)	27.8	4.9
B2 (0.35ppm)	19.4	0.5
#4 (0.48ppm)	180.0	13.4
#4 (2.42ppm)	600.1	81.9
BPA (20ppm)	482.0	39.0

	ng B/10^6 cell	
Sample ID	Av.	SD
control	16.4	2.8
B1 (0.7ppm)	37.8	0.2
B2 (0.7ppm)	31.5	0.4
#4 (0.48ppm)	295.4	4.0
BPA (20ppm)	452.3	6.6

Figure S8. Boron concentrations in SCCVII cells and in U87 cells determined by ICP-OES with standard deviation values.

<u>Calculation of ^{10}B concentration in Medium</u>

1. <u>B1 and B2 : 6 mg/ml DMSO stock solution. If the medium final concentration is 2 micro L/ml, the medium include 0.012 mg/ml of B1 or B2. In 1 mol of B1 or B2 (MW 450.32), ^{10}B weighs 26.4 (22 % of natural boron) g in 450.32 g. Therefore, the medium includes 0.703 ppm (=0.012*26.4/450.32 mg/ml) of ^{10}B.</u>

2. <u>Compound #**4**: 12mg/ml DMSO stock solution. If the medium final concentration is 1 micro L/ml, the medium include 0.012mg/ml of #4. In 1 mol of #4 (MW 652.19), ^{10}B weighs 26.4 (22 % of natural boron) g in 652.19 g. Therefore, the medium includes 0.485ppm (=0.012*26.4/652.19 mg/ml)) of ^{10}B.</u>
